# Inflammatory signaling pathways in neutrophils: implications for breast cancer therapy

**DOI:** 10.1097/MS9.0000000000003251

**Published:** 2025-04-04

**Authors:** Emmanuel Ifeanyi Obeagu, Syed A. A. Rizvi

**Affiliations:** a Department of Biomedical Laboratory Science, Africa University, Zimbabwe; b College of Biomedical Sciences, Larkin University, Miami, Florida, USA; c Division of Clinical and Translational Research, Larkin Community Hospital, Miami, Florida, USA

**Keywords:** breast cancer, inflammatory signaling pathways, neutrophils, therapeutic targeting, tumor microenvironment

## Abstract

Neutrophils, key components of the innate immune system, have emerged as pivotal players in the tumor microenvironment (TME), particularly in breast cancer. These versatile cells contribute to both pro-tumorigenic and anti-tumorigenic processes through inflammatory signaling pathways that influence tumor progression, immune evasion, and therapeutic responses. Their recruitment to the TME, mediated by chemokines such as CXCL1 and CXCL8, and their subsequent activation underscore their complex role in breast cancer biology. Neutrophil extracellular traps, cytokine secretion, and reactive oxygen species production further highlight their dualistic nature in cancer pathophysiology. Critical inflammatory signaling pathways, including nuclear factor kappa-light-chain-enhancer of activated B cells (NF-κB), Janus kinase/signal transducer and activator of transcription (JAK/STAT), mitogen-activated protein kinase (MAPK), and phosphatidylinositol 3-kinase (PI3K)/AKT, regulate neutrophil activity in breast cancer. Dysregulation of these pathways can lead to the promotion of angiogenesis, immune suppression, and metastasis. For example, the NF-κB pathway fosters the secretion of pro-inflammatory cytokines, while JAK/STAT signaling drives the differentiation of tumor-associated neutrophils. The MAPK and PI3K/AKT pathways influence neutrophil survival and chemotactic responses, further enhancing their contribution to the tumor-supportive microenvironment. Understanding these mechanisms provides a framework for therapeutic intervention.

## Introduction

Breast cancer is a type of cancer that develops in the cells of the breast, commonly originating in the ducts or lobules. It can affect both women and men, though it is far more prevalent in women. Key risk factors include genetic predispositions (e.g., BRCA1 or BRCA2 gene mutations), hormonal influences, lifestyle factors, and age. It is the most prevalent malignancy among women worldwide, posing a significant burden on global health systems. Advances in understanding the tumor microenvironment (TME) have revealed the critical roles of immune cells in modulating cancer progression and therapy outcomes^[1]^. Among these immune cells, neutrophils, traditionally recognized for their roles in acute inflammation and microbial defense, have emerged as key players in cancer biology. Their involvement in inflammatory signaling pathways within the TME adds complexity to breast cancer pathophysiology, highlighting their dual role in promoting or inhibiting tumor progression^[2]^.

**HIGHLIGHTS**

- Neutrophils exhibit dual roles in breast cancer, with their inflammatory pathways influencing tumor progression, angiogenesis, and immune evasion.
- NF-κB, STAT3, MAPK, and TGF-β are critical neutrophil signaling pathways promoting breast cancer progression.
- Neutrophil extracellular traps aid in tumor cell migration, immune evasion, and metastasis in breast cancer.
- Targeting neutrophil-driven inflammation with inhibitors (e.g., STAT3 and TGF-β) shows promise in preclinical studies for breast cancer therapy.
- Challenges include neutrophil heterogeneity and translating findings into clinical strategies.

Neutrophils are among the first responders to inflammatory signals and are rapidly recruited to sites of infection or injury. In the context of cancer, their recruitment is largely driven by chemokines such as CXCL1, CXCL2(IL-2), and CXCL8(IL-8), which are often upregulated in the TME^[3]^. Once activated, neutrophils exhibit a diverse range of functions, from releasing cytokines and chemokines to producing reactive oxygen species (ROS) and forming neutrophil extracellular traps (NETs). These functions, while beneficial in microbial defense, can be co-opted by tumors to support cancer progression, angiogenesis, and immune evasion^[4]^.

Neutrophil-driven inflammatory signaling is mediated by several key pathways, including NF-κB, Janus kinase/signal transducer and activator of transcription (JAK/STAT), mitogen-activated protein kinase (MAPK), and phosphoinositide 3-kinase/protein kinase B (PI3K/AKT)^[5]^. Each of these pathways regulates critical neutrophil functions such as cytokine release, chemotaxis, survival, and interaction with other immune and stromal cells within the TME. Dysregulation of these pathways not only amplifies tumor-promoting activities of neutrophils but also contributes to therapy resistance, underscoring their clinical relevance in breast cancer^[6]^.

Interestingly, the role of neutrophils in breast cancer is highly context-dependent. While certain subsets of neutrophils, such as tumor-associated neutrophils (TANs), are known to enhance tumor progression, others exhibit anti-tumorigenic properties, such as promoting tumor cell lysis and enhancing adaptive immune responses^[7]^. This duality is influenced by factors within the TME, such as cytokine milieu, metabolic state, and interactions with other immune cells. Consequently, understanding the specific roles of neutrophil subsets in breast cancer is essential for devising targeted therapeutic strategies^[8]^.

The contribution of neutrophils to breast cancer progression extends beyond direct tumor interactions. Neutrophils also play a significant role in shaping the TME by remodeling the extracellular matrix (ECM), promoting angiogenesis, and modulating immune cell infiltration^[9]^. These processes are intricately linked to inflammatory signaling pathways, making these pathways attractive targets for therapeutic intervention. For instance, inhibiting NF-κB signaling has been shown to reduce neutrophil-driven angiogenesis, while blocking JAK/STAT signaling can prevent the differentiation of pro-tumorigenic TANs^[10]^.

Targeting inflammatory signaling pathways in neutrophils presents significant opportunities for breast cancer therapy. Neutrophils contribute to tumor growth and metastasis by secreting pro-angiogenic factors such as vascular endothelial growth factor (VEGF) and suppressing adaptive immunity. By disrupting these pathways, cancer progression might be mitigated. Conversely, neutrophils also possess anti-tumor properties, such as releasing cytotoxic agents and promoting immune cell infiltration. Enhancing these beneficial roles could improve treatment outcomes. Modulating key inflammatory mediators, including NF-κB, STAT3, and PI3K, may help reduce the tumor-promoting effects of neutrophils while preserving or amplifying their tumor-fighting functions^[11]^.

### Aim

This review aims to provide a comprehensive overview of the inflammatory signaling pathways in neutrophils and their implications for breast cancer therapy.

### Rationale

Neutrophils, as the most abundant immune cells in circulation, play a pivotal role in both innate immunity and tumor dynamics. In the context of breast cancer, their presence within the TME has been associated with both tumor-promoting and tumor-suppressing activities. This duality is mediated by inflammatory signaling pathways, such as NF-κB, JAK/STAT, MAPK, and PI3K/AKT, which orchestrate neutrophil recruitment, activation, and polarization^[12]^.

While significant advancements have been made in understanding the contributions of immune cells to cancer progression, neutrophils have historically been underexplored compared to other immune players like T cells and macrophages. Recent evidence highlighting their critical roles in angiogenesis, metastasis, immune evasion, and therapy resistance underscores the need for a deeper investigation into neutrophil biology in breast cancer. Additionally, the dynamic nature of neutrophils within the TME offers unique opportunities for therapeutic intervention, either by suppressing their pro-tumorigenic functions or enhancing their anti-tumor activities^[13]^.

## Review methods

To ensure a comprehensive and systematic exploration of inflammatory signaling pathways in neutrophils and their implications for breast cancer therapy, we adopted an integrative narrative review methodology. This approach involved an extensive literature search, critical appraisal of selected studies, and synthesis of findings to provide a cohesive discussion on the topic.

### Literature search strategy

A structured search was conducted across multiple electronic databases, including **PubMed, Scopus, Web of Science, and Google Scholar**, to identify relevant peer-reviewed articles published up to the present year. Keywords and Medical Subject Headings terms were utilized to maximize search sensitivity and specificity. The primary search terms included **“neutrophils,” “inflammatory signaling pathways,” “breast cancer,” “tumor-associated neutrophils (TANs),” “NF-κB,” “JAK-STAT,” “PI3K-Akt,” “MAPK,” “immunotherapy,”** and **“tumor microenvironment (TME).”** Boolean operators (AND, OR) were applied to refine the search results.

#### Eligibility criteria

The inclusion and exclusion criteria were defined to ensure the relevance and reliability of the selected studies.

- Inclusion criteria:
  - Studies published in peer-reviewed journals.
  - Original research articles, reviews, and meta-analyses examining neutrophil-mediated inflammatory signaling in breast cancer.
  - Articles focusing on neutrophil signaling pathways (e.g., NF-κB, JAK-STAT, PI3K-Akt, and MAPK) in the TME.
  - Studies discussing therapeutic interventions targeting neutrophil pathways in breast cancer.

- Exclusion criteria:
  - Studies not available in English.
  - Case reports, editorials, and conference abstracts.
  - Studies focusing solely on neutrophils in non-cancerous conditions.
  - Research on other immune cells unless neutrophil interactions were a primary focus.

## Global breast cancer statistics: incidence, mortality, and survival rates

Breast cancer remains a significant global health concern, exhibiting variations in incidence, mortality, and survival rates across different regions and demographics. Understanding these statistics provides insight into the current state of breast cancer and highlights areas needing attention^[8]^.

### Incidence rates

In 2022, breast cancer was diagnosed in approximately 2.3 million women worldwide, making it the most common cancer among women in 157 out of 185 countries.

The incidence of breast cancer varies globally, correlating with socioeconomic factors. In wealthier nations, about 1 in 12 women are diagnosed with breast cancer, whereas in lower-income countries, the ratio is approximately 1 in 27. This disparity is partly attributed to differences in reproductive behaviors, such as age at menarche and childbirth, as well as access to screening and healthcare services^[9]^.

### Mortality rates

Breast cancer caused 670 000 deaths globally in 2022.

Mortality rates also exhibit regional disparities. In wealthier countries, 1 in 71 women succumb to breast cancer, whereas in lower-income countries, the rate is 1 in 48. These differences are largely due to variations in early detection, treatment availability, and healthcare infrastructure^[10]^.

### Survival rates

Survival outcomes are closely linked to the stage at diagnosis. In the United States, the 5-year relative survival rates are as follows:

- localized disease: 99%;
- regional disease: 86%;
- distant-stage disease: 30%.

Globally, survival rates vary, with developed countries generally reporting higher survival due to advanced screening and treatment options. For instance, Australia and New Zealand have the highest breast cancer incidence rates globally, attributed to factors like aging populations and comprehensive screening programs. Despite high incidence, these countries have achieved a 2.1% annual decline in mortality rates, nearing the World Health Organization’s targets^[11^−^12]^.

### Trends and disparities

While breast cancer incidence rates are rising globally, particularly in developing nations, mortality rates have been declining in many high-income countries. This trend is primarily due to improvements in early detection through screening and advancements in treatment. However, disparities persist, with lower survival rates in developing regions, underscoring the need for enhanced healthcare infrastructure, access to screening, and public health initiatives^[13^−^14]^.

## Neutrophils and the tumor microenvironment

Neutrophils, the most abundant circulating white blood cells, are key players in the immune system’s response to infection and injury. However, their functions are complex and context-dependent in the context of cancer. Within the tumor TME, neutrophils can exert both pro-tumorigenic and anti-tumorigenic effects, influenced by the local conditions and inflammatory signals. Their role in cancer progression, particularly in breast cancer, is increasingly recognized as a critical factor that can either promote or inhibit tumor growth, metastasis, and immune evasion^[14]^.

Neutrophils are recruited to the TME through signaling pathways that involve cytokines and chemokines such as CXCL8, granulocyte-colony stimulating factor (G-CSF), and tumor-derived factors like VEGF. Upon entering the TME, neutrophils can undergo polarization, adopting either a pro-inflammatory, tumor-promoting phenotype or an anti-inflammatory, tumor-suppressing phenotype. This polarization is heavily influenced by the microenvironment, particularly the presence of inflammatory cytokines and growth factors. In breast cancer, the polarization of neutrophils toward a pro-tumor phenotype is often associated with an immunosuppressive environment, facilitating tumor growth and metastasis^[15]^.

The pro-tumorigenic neutrophils, often called TANs, support cancer progression in several ways. They can contribute to tumor progression by promoting angiogenesis by producing pro-angiogenic factors like VEGF, matrix metalloproteinases (MMPs), and cytokines such as interleukin (IL)-6 and IL-8. Additionally, TANs can facilitate tumor cell migration and invasion by breaking down the ECM, which supports tumor cell metastasis to distant organs. Neutrophils also play a role in immune suppression within the TME by secreting immunosuppressive cytokines such as transforming growth factor-beta (TGF-β), which dampen the activity of cytotoxic T cells and other immune effectors^[16]^.

Neutrophil extracellular traps are web-like structures composed of DNA, histones, and antimicrobial proteins that neutrophils release in response to infection or injury. While NETs are part of the immune response to pathogens, they have been implicated in cancer progression. NETs can enhance tumor cell proliferation, invasion, and metastasis in breast cancer. These structures can physically trap circulating tumor cells (CTCs) and provide a scaffold that aids their survival, thereby increasing the likelihood of metastasis. Furthermore, NETs contain a variety of enzymes and cytokines that can promote tumor cell migration, invasion, and immune suppression, contributing to an environment conducive to tumor growth^[17]^.

Neutrophils in the TME can also contribute to immune evasion mechanisms that protect the tumor from immune surveillance. For instance, TANs can suppress T-cell activity by producing ROS and releasing immunosuppressive cytokines like TGF-β and IL-10. These molecules inhibit the function of cytotoxic T cells and natural killer (NK) cells, which are critical for tumor eradication. Additionally, neutrophils can induce T cell exhaustion, further dampening anti-tumor immune responses. This immune suppression not only helps the tumor evade immune detection but also creates a vicious cycle that sustains tumor growth^[18]^.

The tumor stroma, composed of fibroblasts, endothelial cells, and the ECM, plays a crucial role in tumor development. Neutrophils contribute to remodeling the tumor stroma by secreting MMPs, which degrade the ECM and facilitate tumor cell migration and metastasis. MMPs like MMP-9 are particularly important in breaking down collagen and other ECM components, allowing tumor cells to invade surrounding tissues. Additionally, neutrophils can influence the composition of the ECM by promoting the secretion of pro-fibrotic factors, which further enhance the remodeling of the TME to support tumor progression^[19]^.

In contrast, neutrophils can also support anti-tumor immunity under certain conditions. When polarized toward an anti-tumor phenotype, neutrophils can promote tumor cell death through phagocytosis and the release of cytotoxic molecules such as ROS and proteases. They can also recruit other immune cells, like dendritic cells (DCs) and cytotoxic T lymphocytes, to the TME, enhancing the overall anti-tumor immune response. The balance between these opposing functions – tumor promoting versus tumor suppressing – largely depends on the inflammatory cues present within the TME and the precise regulation of neutrophil activation and polarization^[20]^.

Neutrophils also interact with other immune cells within the TME, influencing their function and contributing to the overall immune landscape of the tumor. For example, neutrophils can modulate macrophages’ activity by either polarizing them toward a pro-tumor M2 phenotype or activating them into an anti-tumor M1 phenotype. These interactions are complex and depend on the specific signals in the TME, which can either support or inhibit cancer progression. Neutrophils also interact with T cells and NK cells, enhancing their function or promoting exhaustion, as previously discussed. The intricate network of neutrophil–immune cell interactions further underscores the complexity of neutrophil roles in cancer and highlights the need for targeted therapeutic strategies that modulate neutrophil activity^[21]^.

### Recruitment of neutrophils to the TME

Neutrophil recruitment to the TME is mediated by cytokines and chemokines released by tumor cells, stromal cells, and resident immune cells. These signals activate neutrophil integrins and adhesion molecules, enabling their extravasation into the tumor site. The high levels of G-CSF and IL-8 in the TME further enhance neutrophil infiltration. This recruitment is a critical step in neutrophil-driven modulation of the TME, as it sets the stage for their functional activity^[22]^. In the TME, neutrophils undergo polarization into phenotypically distinct subsets with either pro-tumorigenic or anti-tumorigenic properties. Pro-tumorigenic TANs promote angiogenesis, immunosuppression, and ECM remodeling, thereby facilitating tumor progression. The secretion of VEGF, MMPs, and TGF-β characterizes these TANs. Conversely, anti-tumorigenic neutrophils can exert cytotoxic effects on cancer cells through the release of ROS and engagement in antibody-dependent cellular cytotoxicity^[23]^.

A unique mechanism by which neutrophils influence the TME is through the formation of NETs. NETs, composed of DNA, histones, and granule proteins, can trap and kill pathogens; however, in the TME, they have been implicated in promoting metastasis by enhancing tumor cell adhesion and survival in distant organs. NETs also modulate the immune response by inducing an immunosuppressive environment, thereby shielding cancer cells from immune surveillance^[24]^. Neutrophils interact with other immune cells, including macrophages, T cells, and DCs, within the TME. Cytokines, chemokines, and cell surface receptors mediate these interactions. For instance, neutrophils can suppress T cell activity by producing arginase-1 and ROS, leading to an immunosuppressive TME. Additionally, neutrophils can promote macrophage-mediated tumors by secreting chemotactic factors that recruit and polarize macrophages into a tumor-supportive phenotype^[25]^.

Neutrophils play a crucial role in tumor angiogenesis by releasing pro-angiogenic factors such as VEGF and MMP-9. These factors stimulate new blood vessel formation and degrade the ECM, facilitating tumor invasion and metastasis. The ability of neutrophils to modify the ECM further underscores their role in promoting tumor cell dissemination^[26]^. The diverse and context-dependent roles of neutrophils in the TME highlight their complexity as mediators of cancer progression. Their interactions with cancer cells, immune cells, and the ECM create opportunities for therapeutic targeting. However, the dual nature of neutrophil functions necessitates strategies that selectively inhibit their tumor-promoting activities while preserving or enhancing their anti-tumorigenic potential^[27]^.

### Key inflammatory signaling pathways in neutrophils

Neutrophils, essential for the immune response, are regulated by various signaling pathways that govern their activation and function. These pathways ensure neutrophils perform their roles in infection defense and tissue homeostasis. However, in cancer, these pathways can be altered to support tumor growth, metastasis, and immune suppression. Key signaling pathways include the NF-κB, JAK/STAT, MAPK, and PI3K/AKT pathways. The NF-κB pathway regulates inflammation and recruits neutrophils to the TME. In cancer, its dysregulation can promote angiogenesis, immune evasion, and metastasis. The JAK/STAT pathway, activated by cytokines like G-CSF and IL-6, drives neutrophil differentiation into TANs, which often adopt pro-tumorigenic roles. The MAPK pathway responds to growth factors and stress stimuli, regulating neutrophil chemotaxis and cytokine production, further promoting tumor progression. Lastly, the PI3K/AKT pathway controls neutrophil survival and migration, supporting tumor cell survival and metastasis. These pathways highlight neutrophils’ complex role in promoting tumor growth and fighting cancer. Targeting these pathways offers a promising approach to reprogramming neutrophils and enhancing cancer treatment outcomes^[6,28]^.

#### NF-κB pathway

The NF-κB pathway regulates inflammation and immune responses, particularly in neutrophils. This pathway is activated through pattern recognition receptors, such as toll-like receptors (TLRs), which respond to various danger signals, including tumor-derived molecules. Once activated, NF-κB signaling triggers the transcription of several pro-inflammatory cytokines, such as IL-1β and tumor necrosis factor-alpha (TNF-α), as well as chemokines like CXCL1 and CXCL8. These molecules play a crucial role in recruiting and activating neutrophils within the TME. In cancer, dysregulated NF-κB activation in neutrophils contributes significantly to tumor progression. This aberrant activation promotes several pro-tumorigenic processes, such as enhanced angiogenesis, immune evasion, and metastasis. Specifically, NF-κB-induced cytokine production facilitates the recruitment of more immune cells, which can either support or suppress the immune response, depending on the signaling context.

Additionally, the increased secretion of angiogenic factors can help tumors establish the vascular networks they need for growth and survival. Furthermore, NF-κB activation in neutrophils has been shown to contribute to immune evasion by altering immune surveillance mechanisms, making it more difficult for the body’s defenses to recognize and attack cancer cells. Targeting the NF-κB pathway in neutrophils presents a potential strategy for cancer therapy, as it could reduce tumor-promoting inflammation and immune suppression while possibly enhancing anti-tumor immunity. By inhibiting NF-κB signaling, reproducing neutrophils to a more anti-tumorigenic phenotype may be possible, which could improve the effectiveness of existing cancer treatments^[5,29]^.

#### JAK/STAT pathway

The JAK/STAT pathway is a critical signaling mechanism activated by cytokines such as G-CSF and IL-6, which are often found in high concentrations within the TME. This pathway regulates neutrophil biology, including their differentiation, survival, and polarization into TANs. The TANs can exhibit either pro-tumorigenic or anti-tumorigenic functions depending on the signals they receive from the TME, but in many cancers, they are skewed toward a pro-tumorigenic phenotype. Pro-tumorigenic TANs contribute to cancer progression by secretion of various factors that suppress immune surveillance and enhance metastatic processes. One of the pivotal mediators in this pathway is STAT3, a transcription factor that is frequently upregulated in cancer. STAT3 activation drives the expression of genes associated with neutrophil-mediated tumor-supportive activities, including the production of MMPs and VEGF. The MMPs facilitate the breakdown of **ECM** components, promoting tumor invasion and metastasis, while VEGF enhances angiogenesis, supplying the tumor with nutrients and oxygen. The persistent activation of the JAK/STAT pathway in neutrophils within the TME enhances their survival and polarizes them toward a phenotype that actively supports tumor growth and immune evasion. Targeting components of this pathway, such as STAT3, represents a promising therapeutic strategy to disrupt the tumor-supportive functions of neutrophils while potentially reprogramming them to adopt anti-tumorigenic properties. This approach could help restore balance within the TME and improve the efficacy of existing cancer therapies^[30]^.

#### MAPK pathway

The MAPK pathway represents a pivotal signaling axis in neutrophils, triggered by external cues such as growth factors, cytokines, and environmental stressors. This complex pathway comprises three main branches: extracellular signal-regulated kinase (ERK), p38 MAPK, and c-Jun N-terminal kinase (JNK). Each branch has distinct yet interrelated functions, contributing to the regulation of neutrophil activity. ERK primarily facilitates processes associated with cell survival and proliferation. p38 MAPK is critically involved in modulating inflammatory responses and cellular adaptation to stress, while JNK mediates stress-induced apoptosis and other essential cellular mechanisms. In neutrophils, MAPK signaling orchestrates several fundamental activities, including directed migration (chemotaxis), the production and release of cytokines, and the generation of NETs, which are critical in antimicrobial defense. However, in the context of cancer, the MAPK pathway becomes co-opted to enhance neutrophil survival, enabling these cells to persist and participate in processes that inadvertently support tumor growth and progression. This includes promoting angiogenesis, critical for providing nutrients and oxygen to the growing tumor, and suppressing the adaptive immune response, creating a tumor-permissive microenvironment. Through these mechanisms, MAPK signaling sustains neutrophil function and plays a deleterious role in fostering tumor survival and metastasis^[31]^.

#### PI3K/AKT pathway

The PI3K/AKT pathway is crucial for regulating neutrophil survival, metabolism, and migration. In the TME, activation of this pathway supports neutrophil longevity and enhances tumor-promoting functions, such as producing ROS and NETs. ROS contributes to tumor progression by damaging tissues, aiding tumor cell adhesion, and promoting metastasis. NETs can capture cancer cells, facilitating their spread. The PI3K/AKT pathway also interacts with other pathways like NF-κB and MAPK, amplifying inflammatory responses and further supporting tumor growth. Chronic activation of PI3K/AKT in neutrophils can enhance immune suppression, angiogenesis, and metastasis, reinforcing neutrophils’ dual role in supporting and hindering tumor progression. Targeting the PI3K/AKT pathway could offer therapeutic benefits by disrupting neutrophil functions that promote tumor growth while potentially restoring their anti-tumor activity, improving cancer treatment outcomes^[32]^.

### Cross-talk between pathways

The signaling pathways regulating neutrophil functions do not operate independently but are highly interconnected, forming a complex cross-talk network. For example, the NF-κB and STAT3 pathways can synergistically enhance cytokine production, contributing to an inflammatory environment that recruits neutrophils to the TME. At the same time, PI3K/AKT signaling has the ability to modulate the activation of the MAPK pathway, influencing neutrophil migration and effector functions such as the formation of NETs. This interplay among pathways ensures a highly responsive and adaptive immune response. Integrating multiple signaling networks makes understanding how these pathways cooperate in driving neutrophil-mediated inflammation in cancer essential. The cooperative interactions amplify each pathway’s effects, enhancing tumor progression, immune suppression, and metastasis. This complexity highlights the need for targeted therapies that can modulate multiple pathways simultaneously to effectively curb neutrophil-driven inflammation and improve treatment outcomes for cancer patients. Understanding the signaling landscape is crucial for developing therapies to modulate neutrophil behavior to favor anti-tumor immunity while reducing tumor-supportive effects^[6,33]^.

## Therapeutic implications

Targeting neutrophil-mediated inflammatory signaling pathways presents a promising strategy for enhancing breast cancer therapy. Neutrophils, as essential immune system components, exhibit complex behavior in the context of cancer. They can both promote and inhibit tumor growth, depending on their activation state and the TME. This duality is a key aspect that complicates their role in cancer progression. When activated in a pro-tumorigenic environment, neutrophils can release factors that support angiogenesis, metastasis, and immune suppression, all contributing to tumor growth. Pathways such as NF-κB, JAK/STAT, MAPK, and PI3K/AKT are crucial in driving these pro-tumorigenic functions. However, neutrophils can also exert anti-tumor responses in some contexts, such as by enhancing cytotoxic activity or aiding immune cell infiltration into tumors. Understanding and manipulating the signaling pathways that govern these responses holds significant therapeutic potential. By targeting these inflammatory signaling networks, it may be possible to suppress neutrophil functions that support tumor progression while enhancing their anti-tumor capabilities. This can be achieved through various approaches, such as inhibiting specific pro-inflammatory cytokines, modulating the activation of key signaling molecules like STAT3 or NF-κB, or reprogramming neutrophils to adopt a more anti-tumorigenic phenotype. Such interventions could improve therapeutic outcomes, potentially increasing the effectiveness of treatments like chemotherapy or immunotherapy. The strategic modulation of neutrophil activity represents a multifaceted approach to cancer treatment, offering a way to manipulate immune responses to shift the balance toward tumor suppression. Continued research into the precise molecular mechanisms underlying neutrophil behavior in the TME is essential for developing targeted therapies that harness the full potential of these cells in combating breast cancer^[14,34]^.

### Targeting pro-tumorigenic neutrophil functions

Pro-tumorigenic neutrophils, such as TANs, contribute to cancer progression through various mechanisms, including promoting angiogenesis, suppressing anti-tumor immunity, and facilitating metastasis. These cells are typically recruited into the TME, where they secrete factors that support tumor growth and spread. For example, TANs secrete angiogenic factors like VEGF that promote the formation of new blood vessels, thus enhancing tumor nutrient supply. They also produce MMPs, enzymes that degrade the ECM and enable cancer cells to invade surrounding tissues and metastasize. Therapeutic strategies targeting TANs aim to disrupt their pro-tumor functions by reducing their recruitment to the TME or inhibiting their inflammatory activities. One approach involves blocking the chemokines that mediate neutrophil migration to tumors, such as CXCL8 (also known as IL-8). By inhibiting the signaling pathways that lead to neutrophil infiltration, these strategies can reduce the number of TANs in the TME, thus potentially impairing tumor progression. Another potential strategy is to target G-CSF, which plays a critical role in activating and mobilizing neutrophils, including TANs^[35]^. By inhibiting G-CSF or its receptors, it is possible to reduce neutrophil recruitment to the tumor and limit their tumor-promoting activities.

Additionally, targeting key signaling pathways within TANs, such as NF-κB and STAT3, offers another therapeutic avenue. NF-κB and STAT3 are pivotal regulators of inflammation and immune responses, and their activation in TANs is linked to the production of pro-tumorigenic factors, including VEGF and MMPs. Inhibiting these pathways can suppress the secretion of these factors, thus potentially reducing tumor angiogenesis, inhibiting invasion, and decreasing metastasis. As a result, targeting these pathways not only diminishes the ability of TANs to support tumor growth but may also improve the effectiveness of other cancer therapies by reprogramming neutrophils to enhance anti-tumor immunity. Overall, the therapeutic targeting of TANs and their associated signaling pathways represents a promising strategy for limiting tumor progression and enhancing the efficacy of cancer treatments. Through a combination of blocking neutrophil recruitment and inhibiting the pro-tumor functions of these cells, it is possible to reshape the TME and improve clinical outcomes^[36]^.

### Enhancing anti-tumor neutrophil functions

Therapeutic approaches focused on reprogramming neutrophils aim to enhance their anti-tumor functions, shifting their role from tumor-promoting to tumor-fighting. One strategy involves using cytokines, such as interferon-gamma (IFN-γ), which can polarize neutrophils toward an anti-tumor phenotype. This polarization encourages neutrophils to perform cytotoxic activities, directly attacking tumor cells. Additionally, NETs typically associated with tumor progression can be harnessed for targeted drug delivery within the TME. This approach uses the trapping abilities of NETs to direct therapeutic agents precisely to the site of cancer cells, increasing treatment efficacy. By understanding the mechanisms that regulate neutrophil polarization and NET formation, researchers can design therapies that maximize the anti-tumor potential of neutrophils. This could involve enhancing the activation of anti-tumorigenic pathways while inhibiting those that promote inflammation or support tumor growth. Ultimately, reprogramming neutrophils offers a promising avenue for cancer treatment, potentially turning these immune cells into powerful allies against tumor progression^[14,37]^.

### Inhibiting neutrophil recruitment

Neutrophil recruitment to the TME is a critical step in promoting cancer progression, as neutrophils can be recruited by chemokines and cytokines secreted by both tumor and stromal cells. One of the key chemokine receptors involved in neutrophil chemotaxis is CXCR2, which binds to its ligand CXCL8 (also known as IL-8). This signaling axis plays a significant role in driving the infiltration of neutrophils into tumors, where they can then contribute to tumor promotion through various mechanisms, such as immune suppression, angiogenesis, and metastasis. Targeting the CXCR2/CXCL8 axis has shown promise in preclinical studies as a potential therapeutic strategy. By blocking this pathway, the recruitment of neutrophils to the TME can be reduced, limiting their tumor-supporting roles.

Furthermore, inhibiting this axis has been found to alleviate immunosuppression within the TME, thereby facilitating the activation and function of T cells. This is particularly important because T cells play a crucial role in anti-tumor immunity, and neutrophil-mediated immune suppression can impair their effectiveness. By disrupting neutrophil recruitment, therapies targeting CXCR2/CXCL8 may enhance the immune response against the tumor and improve the efficacy of other cancer treatments, including immunotherapies. As research continues to explore the therapeutic potential of targeting neutrophil chemotaxis, it offers a promising approach to reprogramming the TME and reducing the pro-tumorigenic functions of neutrophils^[38]^.

### Combining neutrophil-targeted therapies with immunotherapies

Integrating neutrophil-targeted therapies with immune checkpoint inhibitors (ICIs) has emerged as a promising strategy to enhance cancer treatment outcomes. Combining therapies that modulate neutrophil functions with checkpoint blockade has shown synergistic effects in preclinical models. Neutrophils, particularly TANs, play a significant role in creating an immunosuppressive TME by secreting factors that inhibit T cell activation and promote tumor progression. By targeting these pro-tumorigenic neutrophil activities, it is possible to reduce the immunosuppressive signals within the TME, thus allowing ICIs to function more effectively. For example, inhibiting signaling pathways such as STAT3 in TANs can disrupt the immunosuppressive signals they produce, such as the secretion of cytokines and chemokines that impede T-cell function.

This restoration of T cell activity enhances the efficacy of ICIs, particularly those targeting the PD-1/PD-L1 pathway. PD-1/PD-L1 inhibitors block the interaction between the PD-1 receptor on T cells and its ligand, PD-L1, expressed on tumor cells and immune cells in the TME. This inhibition can boost anti-tumor immune responses by reinvigorating exhausted T cells. However, when neutrophils continue to suppress T cell function, the effectiveness of these inhibitors can be limited. Therefore, combining neutrophil-targeted therapies with ICIs helps reduce the tumor-supportive roles of neutrophils and improves the anti-tumor immune response by allowing for a more pro-inflammatory TME. The combination of neutrophil modulation and checkpoint inhibition holds great therapeutic potential, as it addresses both the immune evasion mechanisms of the tumor and the suppressive factors present in the TME. By targeting multiple pathways involved in immune regulation, these combination strategies may lead to more effective cancer therapies and improved clinical outcomes^[39]^.

### Challenges in therapeutic development

Despite the promising preclinical data showing the potential of neutrophil-targeted therapies in cancer treatment, translating these findings into clinical practice remains challenging. One of the key difficulties lies in the dual nature of neutrophil functions. Neutrophils can exhibit pro-tumorigenic and anti-tumorigenic properties depending on their activation state and the context within the TME. This makes it difficult to develop therapies that specifically target neutrophils’ harmful, tumor-promoting activities without impairing their essential immune functions, which are critical for defending against infections and maintaining tissue homeostasis. Moreover, the heterogeneity of neutrophils adds another layer of complexity. Neutrophils are not a uniform population; their phenotype and function can vary significantly based on their environment, such as the inflammatory signals they receive from the TME. Some neutrophils may promote immune suppression and tumor progression, while others may help mount an anti-tumor immune response. This variability necessitates personalized treatment approaches that can selectively modulate neutrophil behavior in different patients and tumor types^[40]^.

Identifying reliable biomarkers to predict neutrophil activity and their response to therapy is crucial for optimizing these treatment strategies. Such biomarkers could help guide the development of more targeted therapies, allowing clinicians to monitor and adjust treatment regimens based on the specific neutrophil activity in a given patient’s tumor. This could enhance the effectiveness of neutrophil-targeted treatments while minimizing potential side effects. As a result, while neutrophil-targeted therapies offer great promise, significant research is still required to address these challenges, including a better understanding of the mechanisms driving neutrophil polarization and the development of strategies for selectively modulating neutrophil functions within the complex TME^[41]^.

## Advances in drug delivery and nanotechnology

Advancements in drug delivery systems, particularly those utilizing nanoparticle-based approaches, have significantly enhanced the ability to target neutrophil-mediated pathways in cancer therapy^[42]^. Nanoparticles, or nanocarriers, can be precisely engineered to deliver therapeutic agents directly to neutrophils or the TME, reducing systemic toxicity and minimizing off-target effects. This level of specificity is critical, as it allows for targeted modulation of neutrophil activity without disrupting other vital immune functions. Nanoparticles can be tailored to recognize and bind to specific receptors or proteins expressed on neutrophils, ensuring therapeutic agents are efficiently delivered to the correct location. For instance, nanoparticles loaded with inhibitors targeting key signaling pathways, such as NF-κB or PI3K/AKT, have shown promise in modulating neutrophil behavior within the TME. Inhibiting the NF-κB pathway, a critical regulator of inflammation can suppress the production of pro-tumorigenic factors like VEGF and MMPs by neutrophils, reducing tumor growth and metastasis. Similarly, targeting the PI3K/AKT pathway can help alter neutrophil survival and migration, inhibiting their pro-tumorigenic functions while preserving their immune response capabilities^[43]^.

This targeted approach offers significant advantages over traditional therapies by improving the specificity of drug delivery, ensuring that the therapeutic agents are concentrated where they are most needed. Furthermore, the use of nanoparticles can facilitate the controlled release of the drug, allowing for sustained therapeutic effects and reducing the frequency of treatment. As research in this area continues to evolve, nanoparticle-based drug delivery systems can revolutionize cancer therapies, particularly by enhancing the efficacy of treatments that target the immune system and modulate neutrophil functions^[44]^.

## Recent advancements and discoveries in neutrophil biology in breast cancer

Neutrophils, once regarded primarily as short-lived first responders of the innate immune system, have now emerged as pivotal regulators of tumor progression, metastasis, and response to therapy in breast cancer. Over the past decade, significant advancements in neutrophil biology have revealed their remarkable plasticity and diverse functional roles within the TME. Recent discoveries have provided deeper insights into neutrophil heterogeneity, their dynamic interaction with tumor cells, and their impact on immune evasion, angiogenesis, and therapy resistance. These findings have paved the way for novel therapeutic strategies aimed at modulating neutrophil function to improve breast cancer treatment outcomes.

### Neutrophil heterogeneity in breast cancer

Traditionally, neutrophils were thought to be a homogenous population with a singular role in immune defense. However, emerging evidence suggests that TANs exist as a spectrum of functionally distinct subpopulations. These subtypes are largely classified into **N1 (anti-tumorigenic) and N2 (pro-tumorigenic) neutrophils**, though more nuanced classifications are now being proposed.

- **N1 neutrophils** are characterized by their ability to promote anti-tumor immune responses. They exhibit enhanced production of ROS, increased tumor cytotoxicity, and upregulation of pro-inflammatory cytokines such as TNF-α and IFN-γ.
- **N2 neutrophils**, on the other hand, support tumor progression by promoting angiogenesis, secreting immunosuppressive cytokines (IL-10, TGF-β), and suppressing the activity of cytotoxic T cells and NK cells.

Recent single-cell RNA sequencing (scRNA-seq) studies have provided a more detailed characterization of TAN subsets. For instance, a study published in *Nature Cancer* identified transcriptionally distinct neutrophil clusters with specific roles in tumor progression, therapy resistance, and immune modulation. These findings highlight the need for refined biomarkers to distinguish pro-tumorigenic from anti-tumorigenic neutrophil populations^[45,46]^.

### The role of neutrophil extracellular traps (NETs) in breast cancer progression

Neutrophil extracellular traps are web-like structures composed of DNA, histones, and antimicrobial proteins that neutrophils release to trap and neutralize pathogens. Recent studies have demonstrated that NETs also play a critical role in breast cancer progression by enhancing metastasis and immune evasion.

- NETs create a scaffold that facilitates **tumor cell adhesion and invasion**, providing a favorable niche for CTCs to establish metastatic lesions.
- Tumor-derived factors, such as G-CSF and IL-8, can induce neutrophil NETosis, promoting an inflammatory and immunosuppressive environment.
- NET-associated proteases, including neutrophil elastase and myeloperoxidase, contribute to ECM remodeling, further facilitating tumor invasion and dissemination.

Recent discoveries have also linked NETs to **chemotherapy resistance in breast cancer**. A study in *Science Translational Medicine* found that NETs protect tumor cells from apoptosis induced by chemotherapy, suggesting that targeting NET formation could enhance treatment efficacy. The development of NET inhibitors, such as **PAD4 (peptidylarginine deiminase 4) inhibitors**, has shown promise in preclinical models by reducing metastasis and sensitizing tumors to therapy^[47,48]^.

### Neutrophil metabolism and tumor microenvironment interactions

Recent research has shed light on the metabolic adaptations of neutrophils within the TME and how these alterations influence tumor progression. Unlike their classical glycolytic metabolism, TANs exhibit **enhanced lipid metabolism**, particularly fatty acid oxidation (FAO), which sustains their survival and function in the TME.

- Hypoxic and nutrient-deprived conditions within the breast cancer TME reprogram neutrophil metabolism to enhance their **immunosuppressive** capabilities.
- Lipid-rich environments promote the differentiation of neutrophils toward an N2-like phenotype, supporting tumor growth and immune evasion.
- Increased oxidative metabolism in TANs correlates with higher levels of **programmed death-ligand 1 (PD-L1)** expression, further suppressing anti-tumor immunity.

Targeting metabolic pathways in neutrophils has been proposed as a novel therapeutic strategy. Inhibitors of FAO, such as **etomoxir**, have shown potential in reversing neutrophil-mediated immune suppression and restoring anti-tumor immunity.

### Neutrophils and breast cancer immunotherapy resistance

One of the most significant challenges in breast cancer treatment is the limited efficacy of ICIs, such as anti-PD-1 and anti-cytotoxic T-lymphocyte-associated protein 4 (CTLA-4) therapies. Recent findings suggest that neutrophils contribute to this resistance through multiple mechanisms.

- **Neutrophil-mediated T cell suppression**: TANs express high levels of PD-L1, arginase-1 (Arg-1), and ROS, which collectively inhibit T cell proliferation and activation.
- **Exosome-mediated immune suppression**: Neutrophils release **tumor-promoting exosomes** containing microRNAs (miRNAs) (e.g., miR-223 and miR-155) that suppress anti-tumor immune responses.
- **CXCR2 signaling and ICI resistance**: CXCR2, a chemokine receptor highly expressed on neutrophils, plays a crucial role in recruiting TANs to tumors. Studies have shown that **CXCR2 inhibition** can enhance the efficacy of ICIs by reducing neutrophil-mediated immune suppression^[47]^.

These findings underscore the potential of **combination therapies**, integrating neutrophil-targeted strategies with immune checkpoint blockade to overcome resistance in breast cancer.

## Emerging therapeutic strategies targeting neutrophils in breast cancer

Given the central role of neutrophils in breast cancer progression, several therapeutic strategies are being explored to target their tumor-promoting functions:

### CXCR2 inhibitors

- **CXCR2 antagonists (e.g., SX-682 and AZD5069)** prevent neutrophil trafficking to tumors and have shown promising results in preclinical breast cancer models.
- Clinical trials are evaluating the combination of CXCR2 inhibitors with ICIs to enhance anti-tumor immunity.

### NET inhibitors

- **PAD4 inhibitors (e.g., Cl-amidine)** prevent NET formation and reduce metastasis.
- **DNase I therapy** has been explored as a strategy to degrade NETs and reduce their tumor-promoting effects.

### Metabolic modulators

- **FAO inhibitors (e.g., etomoxir)** reprogram neutrophils away from an immunosuppressive phenotype.
- **Glucose metabolism inhibitors** can prevent neutrophil-driven therapy resistance.

### Neutrophil-targeted nanoparticles

- Recent advances in **nanotechnology** have enabled the development of nanoparticles that selectively deliver therapeutic agents to neutrophils.
- **Neutrophil-mimicking biomaterials** are being designed to reprogram the TME by modulating inflammatory signaling^[48]^.

## Inflammatory signaling pathways in neutrophils: regulation, cross-talk, and specific roles in breast cancer

Neutrophils play a central role in orchestrating inflammatory responses, and their function within the TME is heavily influenced by intricate signaling pathways. These pathways regulate neutrophil recruitment, activation, differentiation, and plasticity, shaping their pro- or anti-tumorigenic roles. In breast cancer, specific inflammatory signaling cascades – including the **NF-κB, JAK-STAT, PI3K-Akt, TGF-β, and CXCR2** pathways – govern neutrophil behavior, modulating immune suppression, angiogenesis, metastasis, and therapy resistance. A deeper understanding of these pathways and their cross-talk is critical for identifying novel therapeutic strategies that target tumor-promoting neutrophil functions while preserving their protective roles^[49,50]^.

### NF-κB pathway: a central regulator of neutrophil-mediated inflammation in breast cancer

The **nuclear factor kappa-light-chain-enhancer of activated B cells (NF-κB)** pathway is a master regulator of inflammation and immune responses in neutrophils. This signaling cascade is activated by pro-inflammatory cytokines such as **TNF-α**, IL-1 beta (**IL-1β**), and pathogen-associated molecular patterns (PAMPs) via **TLRs**.

- **Activation mechanism**:NF-κB activation is initiated when stimuli such as TNF-α or IL-1β bind to their respective receptors, leading to the phosphorylation of **IκBα (inhibitor of NF-κB)** by the IKK complex (IκB kinase). This phosphorylation triggers IκBα degradation, allowing NF-κB to translocate into the nucleus and promote the transcription of pro-inflammatory genes.
- **Role in the tumor microenvironment**:In breast cancer, NF-κB signaling in neutrophils promotes the secretion of **VEGF** and **MMPs**, both of which facilitate tumor angiogenesis and ECM remodeling. Additionally, NF-κB activation enhances neutrophil survival and differentiation toward a **pro-tumorigenic N2 phenotype**, leading to the suppression of cytotoxic T cells through the release of immunosuppressive cytokines like **IL-10** and **TGF-β**.
- **Cross-talk with other pathways**:NF-κB signaling interacts with the **JAK-STAT** pathway to modulate neutrophil plasticity. Additionally, NF-κB-driven inflammation can activate the **PI3K-Akt** pathway, further supporting neutrophil survival and expansion within the TME^[9,51]^.

### JAK-STAT pathway: modulating neutrophil polarization in breast cancer

The **JAK-STAT** pathway is a key signaling cascade involved in neutrophil function, particularly in response to cytokines such as **G-CSF, IL-6**, and **IL-10**.

- **Activation mechanism**:Ligand binding to cytokine receptors leads to JAK phosphorylation, which, in turn, phosphorylates and activates STAT proteins. In neutrophils, **STAT3** and **STAT5** are the primary transcription factors mediating tumor-promoting effects.
- **Role in the tumor microenvironment:**
  - **STAT3 activation** enhances the immunosuppressive capacity of neutrophils by upregulating **arginase-1 (Arg-1)**, an enzyme that depletes l-arginine, thereby suppressing T-cell proliferation.
  - **STAT5 signaling** supports neutrophil survival and differentiation into the N2 phenotype, further promoting tumor immune evasion.
  - **G-CSF-induced JAK-STAT activation** increases neutrophil recruitment to the tumor site, where they contribute to inflammation and metastasis.
- **Cross-talk with other pathways**: The JAK-STAT pathway is closely linked with **TGF-β signaling**, which enhances neutrophil-mediated immunosuppression. Additionally, STAT3 activation promotes the expression of NF-κB target genes, thereby sustaining chronic inflammation in the TME^[52,53]^.

### PI3K-Akt pathway: supporting neutrophil survival and tumor progression

The **phosphatidylinositol 3-kinase (PI3K)-Akt** pathway is crucial for regulating neutrophil survival, metabolism, and migration. In breast cancer, this pathway is frequently hyperactivated, reinforcing neutrophil-mediated immune evasion and angiogenesis.

- **Activation mechanism**:The binding of cytokines (e.g., **IL-8, CXCL1**, and **CXCL2**) or growth factors (e.g., **VEGF** and **G-CSF**) to receptor tyrosine kinases (RTKs) or G-protein-coupled receptors (GPCRs) activates PI3K, leading to phosphorylation and activation of Akt.
- **Role in the tumor microenvironment**:
  - PI3K-Akt signaling enhances neutrophil **survival** by inhibiting apoptosis through **Bcl-2 upregulation**.
  - It promotes the secretion of **pro-angiogenic factors**, including **VEGF and MMP-9**, facilitating tumor growth and metastasis.
  - Inhibiting **PI3Kγ**, a neutrophil-specific isoform of PI3K, has been shown to **reprogram immunosuppressive neutrophils into an anti-tumor phenotype**, restoring T cell function.
- **Cross-talk with other pathways**:PI3K-Akt interacts with **NF-κB signaling** to promote sustained neutrophil activation. Additionally, it regulates mammalian target of rapamycin (**mTOR signaling**), influencing neutrophil metabolism and function within hypoxic tumor regions^[54,55]^.

### TGF-β signaling: driving neutrophil-mediated immune suppression

The **TGF-β** pathway is a well-established immunosuppressive axis in cancer. TGF-β is abundantly produced in breast cancer TME and plays a crucial role in shaping neutrophil behavior.

- **Activation mechanism**:TGF-β binds to its receptor (TGF-βR), triggering the phosphorylation of **SMAD2/3**, which then forms a complex with SMAD4 and translocates to the nucleus to regulate gene transcription.
- **Role in the tumor microenvironment**:
  - TGF-β signaling skews neutrophil differentiation toward the **N2 phenotype**, enhancing their tumor-promoting effects.
  - It suppresses **neutrophil-mediated cytotoxicity** by downregulating ROS production.
  - TGF-β-exposed neutrophils contribute to T-cell suppression by increasing the expression of **PD-L1**, thereby promoting immune evasion.
- **Cross-talk with other pathways**:TGF-β signaling cooperates with **JAK-STAT** and **PI3K-Akt** pathways to reinforce neutrophil-mediated tumor progression^[55]^.

### CXCR2 signaling: regulating neutrophil recruitment and therapy resistance

The **C-X-C motif chemokine receptor 2 (CXCR2)** pathway is a key driver of neutrophil recruitment into the breast cancer TME.

- **Activation mechanism**:CXCR2 is activated by its ligands, **CXCL1, CXCL2, CXCL5**, and **CXCL8 (IL-8)**, leading to neutrophil chemotaxis.
- **Role in the tumor microenvironment**:
  - CXCR2 signaling promotes **neutrophil infiltration**, reinforcing inflammation and immune suppression.
  - It enhances **resistance to ICIs** by sustaining neutrophil-mediated T-cell suppression.
- **Cross-talk with other pathways**:CXCR2 signaling intersects with **NF-κB** and **PI3K-Akt**, amplifying pro-tumorigenic inflammatory responses^[56]^.

## Clinical translation of neutrophil-targeted therapies in breast cancer: current advances, emerging technologies, and challenges

Neutrophils have emerged as key regulators of tumor progression, immune evasion, and therapy resistance in breast cancer. Given their dual role in cancer – both tumor-promoting and immune-supportive – there has been a growing interest in targeting neutrophil-associated signaling pathways as a therapeutic strategy. Several ongoing clinical trials and novel therapeutic technologies aim to modulate neutrophil function, by blocking their recruitment, reprogramming them into anti-tumor phenotypes, or preventing their immunosuppressive activities. However, translating these findings into effective clinical interventions presents significant challenges, including heterogeneity in neutrophil function, dynamic plasticity, and potential adverse effects on host immunity. This discussion explores recent advancements in neutrophil-targeted therapies, updates on clinical trials, emerging technologies, and the challenges in bringing these discoveries into clinical practice^[8,57]^.

### Ongoing clinical trials targeting neutrophils in breast cancer

Clinical trials investigating neutrophil-modulating therapies have gained momentum, with several approaches currently being explored to inhibit tumor-promoting neutrophil functions or enhance their anti-cancer activity.

- **CXCR2 antagonists to block neutrophil recruitment:**One of the most promising approaches involves targeting the **C-X-C motif chemokine receptor 2 (CXCR2)**, which mediates neutrophil recruitment into the TME. Several clinical trials are evaluating CXCR2 inhibitors as a means to reduce neutrophil-mediated immune suppression:
  - **AZD5069**, a CXCR2 antagonist, is currently under investigation in combination with ICIs in patients with advanced solid tumors, including breast cancer (**NCT04123379**). Early findings suggest that blocking CXCR2 improves T-cell infiltration and enhances the efficacy of anti-PD-1 therapies.
  - Another CXCR2 inhibitor, **SX-682**, is being tested in a phase I trial (**NCT03161431**) for metastatic breast cancer, particularly in patients resistant to standard immunotherapies.
- **PI3K inhibitors for reprogramming neutrophils:**The **PI3Kγ isoform** is predominantly expressed in myeloid cells, including neutrophils, and plays a crucial role in their immunosuppressive function. Targeting PI3Kγ can reprogram TANs to restore anti-tumor immunity.
  - The PI3Kγ inhibitor **IPI-549** is currently being evaluated in combination with ICIs in a phase II trial (**NCT03961698**) for patients with triple-negative breast cancer (TNBC). Preliminary data suggest that PI3K inhibition can shift neutrophils from a pro-tumorigenic to an anti-tumor phenotype.
- **TGF-β inhibitors to prevent neutrophil-mediated immunosuppression:**Since **TGF-β** skews neutrophils toward an immunosuppressive N2 phenotype, blocking this pathway is another promising strategy.
  - **Galunisertib (LY2157299)**, a TGF-β inhibitor, is currently being tested in combination with chemotherapy and immunotherapy in breast cancer patients (**NCT02423343**). The goal is to reduce neutrophil-mediated immune suppression and improve response rates to standard treatments.
- **Neutrophil depletion strategies using anti-G-CSF therapies:**Given that **G-CSF** plays a role in neutrophil expansion and mobilization, G-CSF-neutralizing antibodies are being explored as a strategy to limit neutrophil-driven tumor progression.
  - **Trilaciclib**, a CDK4/6 inhibitor with neutropenia-mitigating effects, is under evaluation for its ability to modulate neutrophil function in breast cancer patients receiving chemotherapy (**NCT02978716**)^[58,59]^.

### Emerging therapeutic technologies for neutrophil modulation

Beyond pharmacological inhibitors, novel therapeutic technologies are being developed to manipulate neutrophil function in breast cancer.

- **Nanoparticle-based drug delivery for selective neutrophil targeting:**Nanotechnology offers a promising approach to selectively deliver immunomodulatory agents to neutrophils while minimizing systemic toxicity. **Lipid nanoparticles** and **polymeric nanoparticles** are being designed to encapsulate CXCR2 inhibitors, TGF-β blockers, or PI3Kγ inhibitors, ensuring localized drug release within the TME. Preclinical studies have shown that nanoparticle-mediated delivery enhances drug efficacy and reduces off-target effects.
- **Gene editing strategies using CRISPR/Cas9:**CRISPR-based gene editing is being explored to modify neutrophil function directly. Preclinical models have demonstrated that CRISPR-mediated deletion of **PD-L1** in neutrophils restores T-cell activation and enhances anti-tumor immunity. This approach holds promise for the development of personalized neutrophil-targeted therapies.
- **Cell therapy approaches: engineering anti-tumor neutrophils:**Similar to CAR-T therapy, researchers are investigating methods to **genetically engineer neutrophils** to enhance their cytotoxicity against tumor cells. Early experimental approaches involve modifying neutrophils to produce **IFN-γ** or **granzyme B**, which can enhance their anti-tumor activity^[34,60]^.

### Challenges in translating neutrophil-targeted therapies into clinical practice

Despite promising advances, several key challenges must be addressed before neutrophil-targeted therapies can become standard treatments in breast cancer.

- **Heterogeneity and plasticity of neutrophils:**Neutrophils exhibit **high plasticity**, transitioning between pro-tumorigenic (N2) and anti-tumorigenic (N1) phenotypes depending on the TME. This variability makes it difficult to develop targeted therapies that selectively eliminate tumor-promoting neutrophils without compromising host defense mechanisms.
- **Potential for increased infection risk:**Given their essential role in pathogen defense, targeting neutrophils could increase the risk of infections. Careful patient selection and biomarker-driven approaches are needed to minimize adverse effects.
- **Lack of predictive biomarkers for patient stratification:**Identifying biomarkers that can predict **which patients will benefit** from neutrophil-targeted therapies remains a significant challenge. Ongoing research is focused on developing neutrophil-based biomarkers, such as **CXCR2 expression levels** or **neutrophil-to-lymphocyte ratios (NLRs)**, to guide treatment decisions.
- **Resistance mechanisms and tumor adaptation:**Tumors can adapt to neutrophil-targeted interventions by activating alternative immune evasion pathways. Combination therapies that target multiple pathways simultaneously (e.g., **CXCR2 inhibitors + PD-1 blockade**) are being explored to overcome resistance.
- **Regulatory and ethical considerations in cell-based neutrophil therapies:**While gene editing and cell therapy approaches hold great promise, regulatory approvals for genetically modified neutrophils will require extensive safety and efficacy evaluations.^65^

### Future directions and outlook

The integration of **neutrophil-targeted therapies** into breast cancer treatment regimens represents a paradigm shift in cancer immunotherapy. Future research should focus on:

- **Developing combination strategies** that enhance ICIs by overcoming neutrophil-mediated resistance.
- **Identifying neutrophil-specific biomarkers** for precision medicine approaches.
- **Expanding the role of nanotechnology** to improve drug delivery and minimize off-target effects.
- **Advancing clinical trials** that incorporate real-time monitoring of neutrophil dynamics to optimize treatment responses.

## New perspectives and innovative hypotheses in neutrophil biology for breast cancer therapy

The role of neutrophils in breast cancer remains a complex and evolving field of study, with recent discoveries highlighting their dual functions in tumor progression and immune regulation. Despite the growing interest in neutrophil biology, several key gaps persist in our understanding of how these cells interact with the TME, contribute to therapy resistance, and influence patient outcomes. To bridge these gaps, new perspectives and innovative hypotheses must be explored to uncover potential therapeutic targets and redefine neutrophil-based interventions. This discussion introduces novel angles on neutrophil heterogeneity, metabolic reprogramming, extracellular vesicle (EV)-mediated communication, epigenetic regulation, and the potential role of engineered neutrophils in breast cancer therapy.

### Re-evaluating neutrophil heterogeneity: are there more than just N1 and N2?

Traditionally, neutrophils have been categorized into **pro-tumorigenic (N2) and anti-**tumorigenic (N1) phenotypes, with the former supporting cancer progression through immunosuppression, angiogenesis, and metastasis, while the latter enhances immune-mediated tumor clearance. However, emerging evidence suggests that neutrophil subpopulations are far more diverse than this binary classification^[45]^.

*Hypothesis: The existence of functionally distinct neutrophil subsets in breast cancer*

We propose that neutrophils within the breast cancer TME exist along a spectrum of functional states, influenced by tumor stage, immune signals, metabolic conditions, and therapy **exposure**. Single-cell RNA sequencing (scRNA-seq) and proteomic analyses could uncover previously unidentified neutrophil subsets with distinct gene expression profiles and functional roles. For example, pro-inflammatory neutrophils (N1-like) that promote T-cell activation may coexist with immunosuppressive neutrophils (N2-like) that inhibit CD8 + responses, while additional subsets may specialize in tumor invasion, ECM remodeling, or chemoresistance^[61]^.

### Neutrophil metabolism as a target for breast cancer therapy

Recent advances in cancer immunometabolism have revealed that neutrophils undergo metabolic reprogramming in response to signals from the TME. Unlike resting neutrophils, which primarily rely on glycolysis, TANs demonstrate enhanced mitochondrial respiration, lipid metabolism, and amino acid utilization, contributing to their immunosuppressive functions.

*Hypothesis: targeting neutrophil metabolism to reprogram tumor-promoting functions*

We propose that inhibiting specific metabolic pathways in neutrophils could reprogram them from an immunosuppressive to an immune-supportive phenotype. For instance:

- **Lipid metabolism inhibitors** (e.g., FAO blockers) may reduce neutrophil-mediated T-cell suppression.
- **Mitochondrial uncoupling agents** could disrupt the oxidative phosphorylation necessary for N2 neutrophil survival.
- **Glutamine metabolism inhibitors** might impair neutrophil-mediated angiogenesis.

By targeting metabolic dependencies unique to tumor-promoting neutrophils, new therapeutic strategies could emerge to shift the balance toward an anti-tumor immune response.

### Extracellular vesicles and neutrophil-mediated tumor communication

Neutrophils communicate with other cells in the TME through the secretion of EVs, including exosomes and microvesicles. These EVs contain pro-tumorigenic cargo such as cytokines, miRNAs, and metabolites that promote immune evasion, angiogenesis, and metastasis. However, their exact role in therapy resistance and metastatic progression remains poorly understood.

*Hypothesis: Neutrophil-derived extracellular vesicles as drivers of chemoresistance and metastatic niches*

We propose that neutrophil-derived EVs serve as vehicles for transferring chemoresistance signals to breast cancer cells.

- **Exosomal miRNAs** (e.g., miR-223, miR-155) from neutrophils may reprogram cancer cells to resist apoptosis following chemotherapy.
- **Lipid-rich microvesicles** from neutrophils may facilitate metastasis by creating a pro-inflammatory pre-metastatic niche.
- **Neutrophil-derived EVs carrying TGF-β and arginase-1** may suppress anti-tumor immune responses and enhance the efficacy of **myeloid-derived suppressor cells** in promoting immune escape.

If validated, targeting neutrophil-derived EVs could represent a **novel therapeutic approach** to disrupt tumor-neutrophil crosstalk and prevent metastasis.

### Epigenetic reprogramming of neutrophils in breast cancer

Neutrophils exhibit remarkable plasticity in response to **tumor-derived signals, inflammatory mediators**, and **metabolic cues**. Recent research suggests that **epigenetic modifications**, including DNA methylation, histone modifications, and chromatin remodeling, play a key role in shaping neutrophil function. However, the extent to which **epigenetic reprogramming** contributes to neutrophil-mediated tumor progression remains largely unexplored.

*Hypothesis: Epigenetic modulation as a strategy to reprogram neutrophils for cancer immunotherapy*

We propose that epigenetic **inhibitors targeting histone-modifying enzymes [e.g., histone deacetylase (HDAC), DNA methyltransferase (DNMT) inhibitors]** could be used to shift neutrophils from a tumor-promoting to a tumor-suppressive state.

- **Histone deacetylase (HDAC) inhibitors** may prevent the upregulation of immunosuppressive genes in neutrophils.

- **DNA methyltransferase (DNMT) inhibitors** could restore the expression of genes required for neutrophil-mediated tumor clearance.
- **Targeting specific chromatin remodeling factors** may block neutrophil differentiation into an immunosuppressive phenotype.

If successful, epigenetic therapy could offer a **non-cytotoxic strategy** to manipulate neutrophil behavior without depleting their numbers.

### Engineering neutrophils for breast cancer therapy: a future CAR-neutrophil approach?

While chimeric antigen receptor (CAR) T-cell therapy has revolutionized cancer immunotherapy, the potential of **engineered neutrophils** has not yet been explored. Given their natural ability to infiltrate tumors and modulate immune responses, genetically modified neutrophils could be a **next-generation cellular immunotherapy** for breast cancer.

*Hypothesis: CAR-neutrophils as an emerging strategy for targeted immunotherapy*

We propose a **CAR-neutrophil approach**, where neutrophils are genetically engineered to:

- **Express tumor-targeting receptors** to enhance their anti-tumor cytotoxicity.
- **Secrete immune-activating cytokines (e.g., IL-12 and IFN-γ)** to boost T-cell responses.
- **Resist tumor-induced immunosuppression** by knocking out TGF-β or PD-L1 pathways.

Such engineered neutrophils could serve as **“first responders”** in the TME, clearing tumor cells before adaptive immunity is fully activated. Given their short lifespan and high turnover rate, CAR-neutrophils may also **pose fewer safety risks** compared to long-lived CAR-T cells.

## The cytokines and chemokines (apart from CXCL1, CXCL2, and CXCL8) that are involved in the activation and recruitment of neutrophils in the TME in breast cancer

In breast cancer, the TME is a complex ecosystem where various immune cells, cytokines, and chemokines interact to influence cancer progression, immune evasion, and therapeutic responses. Neutrophils, as key players in the inflammatory response, are attracted to the TME by a variety of signaling molecules, including cytokines and chemokines. While CXCL1, CXCL2, and CXCL8 (IL-8) are among the most well-characterized chemokines involved in neutrophil recruitment, other cytokines and chemokines also play significant roles in regulating neutrophil activation and recruitment to the TME.

### CCL2 (monocyte chemoattractant protein-1)

CCL2 is a well-known chemokine involved in the recruitment of various immune cells, including neutrophils, to the TME. It is primarily produced by tumor cells, stromal cells, and immune cells in response to inflammatory stimuli. CCL2 acts by binding to the CCR2 receptor on neutrophils, promoting their migration toward the tumor site. This chemokine has been implicated in promoting tumor progression and metastasis by facilitating the recruitment of both neutrophils and monocytes, which can contribute to an immunosuppressive microenvironment^[62]^.

### IL-1β (interleukin-1 beta)

IL-1β is a pro-inflammatory cytokine that plays a crucial role in neutrophil activation and recruitment. It can be produced by various cell types in the TME, including tumor cells, fibroblasts, and infiltrating immune cells. IL-1β enhances neutrophil survival and activates the release of other pro-inflammatory molecules, including additional cytokines and chemokines, such as IL-6 and TNF-α. IL-1β induces neutrophil migration through the upregulation of adhesion molecules and the secretion of chemokines like CXCL8. Furthermore, IL-1β has been shown to contribute to the development of a chronic inflammatory environment in breast cancer, which can support tumor progression^[63]^.

### IL-17 (interleukin-17)

IL-17 is produced by Th17 cells, a subset of T helper cells, and plays a central role in the regulation of neutrophil recruitment to inflammatory sites. In breast cancer, IL-17 has been shown to stimulate the production of several neutrophil-attracting chemokines, such as CXCL1, CXCL5, and CXCL8. It can also enhance the pro-tumorigenic functions of neutrophils by promoting the secretion of angiogenic factors and MMPs, which facilitate tumor invasion and metastasis. Elevated IL-17 levels in the TME are associated with poor prognosis and increased metastasis in breast cancer^[64]^.

### GM-CSF (granulocyte-macrophage colony-stimulating factor)

GM-CSF is a cytokine that plays a critical role in the activation, differentiation, and survival of neutrophils. It is produced by various cells in the TME, including tumor cells, endothelial cells, and macrophages. GM-CSF can directly enhance the ability of neutrophils to secrete inflammatory mediators and increase their chemotactic response to other cytokines and chemokines. GM-CSF also plays a role in the polarization of neutrophils toward a more pro-tumorigenic phenotype, contributing to tumor progression and metastasis^[65]^.

### TNF-α (tumor necrosis factor-alpha)

TNF-α is a potent pro-inflammatory cytokine involved in the recruitment and activation of neutrophils in the TME. It can be produced by tumor cells, macrophages, and other immune cells. TNF-α can stimulate neutrophil adhesion to endothelial cells, increase vascular permeability, and promote the release of additional pro-inflammatory cytokines and chemokines, including IL-1β and CXCL8. TNF-α also has a dual role in the TME, as it can either promote anti-tumor immune responses or contribute to tumor progression depending on the context. Elevated TNF-α levels in the TME are associated with enhanced angiogenesis, metastasis, and poor patient prognosis in breast cancer^[66]^.

### CXCL5 (C-X-C motif chemokine ligand 5)

CXCL5 is another chemokine that plays an important role in neutrophil recruitment to the TME. Like CXCL1 and CXCL2, CXCL5 acts through the CXCR2 receptor on neutrophils, facilitating their migration toward the tumor site. CXCL5 has been implicated in promoting breast cancer metastasis by enhancing the pro-inflammatory environment within the TME and by stimulating angiogenesis. Increased expression of CXCL5 in breast cancer patients correlates with poor prognosis, highlighting its role in facilitating tumor progression^[67]^.

### CXCL12 (stromal-derived factor-1)

CXCL12 is a chemokine that is primarily involved in the recruitment of immune cells to sites of inflammation. Although it is best known for its role in the recruitment of hematopoietic stem cells, CXCL12 has also been shown to attract neutrophils to the TME. CXCL12 acts through its receptor, CXCR4, which is expressed on neutrophils and tumor cells. In breast cancer, CXCL12 is associated with tumor cell migration, invasion, and metastasis. Neutrophils attracted by CXCL12 can enhance tumor progression by promoting angiogenesis, ECM remodeling, and immune suppression in the TME^[68]^.

### TGF-β (transforming growth factor beta)

TGF-β is a multifunctional cytokine that plays a key role in immune regulation and tumor progression. In the context of neutrophils, TGF-β can promote their recruitment to the TME by inducing the production of chemokines such as CXCL1, CXCL2, and CXCL5. However, TGF-β is also known to induce a suppressive phenotype in neutrophils, converting them into TANs that facilitate immune evasion and tumor progression. TGF-β-induced neutrophils can enhance metastasis by secreting matrix-degrading enzymes and facilitating the epithelial-to-mesenchymal transition of tumor cells^[69]^.

### IL-6 (interleukin-6)

IL-6 is a cytokine that plays a critical role in the activation of neutrophils in the TME. IL-6 promotes neutrophil recruitment by stimulating the production of chemokines such as CXCL8 and CXCL1. It also plays a role in neutrophil survival and activation. In breast cancer, IL-6 is often upregulated in response to tumor cells and inflammatory signals. Elevated IL-6 levels are associated with poor prognosis, tumor progression, and resistance to therapy, as IL-6 can promote an immunosuppressive microenvironment that supports tumor growth^[70,71]^.

## Molecular aspects of how the PI3K/AKT, JAK/STAT, MAPK, and NF-κB pathways affect neutrophil activities in breast cancer

In breast cancer, neutrophils are key components of the TME, where they contribute to tumor progression, metastasis, and immune modulation. Their activation and recruitment are regulated by intricate signaling pathways that control various aspects of their behavior, including migration, survival, degranulation, and the release of pro-inflammatory mediators. Among these, the PI3K/AKT, JAK/STAT, MAPK, and NF-κB pathways have been extensively studied for their roles in neutrophil function within the TME. These pathways influence neutrophil activity through molecular mechanisms that regulate their response to tumor-derived signals and impact the inflammatory environment that can either promote or inhibit cancer progression^[50]^.

### PI3K/AKT pathway

The PI3K/AKT pathway is a central regulator of cell survival, migration, and metabolic processes. In neutrophils, activation of the PI3K/AKT signaling cascade is crucial for their response to chemotactic signals and their survival in the TME. Upon engagement of cell surface receptors such as CXCR1, CXCR2, and IL-8 receptors, the PI3K enzyme is activated, leading to the production of phosphatidylinositol (3,4,5)-trisphosphate (PIP3). This lipid second messenger then recruits and activates AKT, which regulates various downstream targets involved in cell survival, cytoskeletal reorganization, and migration.

In the context of breast cancer, the PI3K/AKT pathway supports neutrophil functions such as:

- **Neutrophil migration**: AKT activation induces cytoskeletal remodeling and facilitates neutrophil migration toward the TME in response to chemokines such as CXCL1, CXCL2, and IL-8.
- **Neutrophil survival**: AKT promotes neutrophil survival by inhibiting apoptotic pathways, which is particularly important in the TME, where prolonged neutrophil survival can contribute to chronic inflammation and tumor progression.
- **Inflammation**: AKT activation can increase the release of pro-inflammatory cytokines, including TNF-α and IL-6, which further amplify the inflammatory environment in the TME.

Furthermore, the PI3K/AKT pathway also plays a role in the polarization of neutrophils into a pro-tumorigenic phenotype. Neutrophils in the TME can adopt an N2-like phenotype, which promotes tumor growth, metastasis, and angiogenesis, partially through PI3K/AKT-mediated signaling^[72]^.

### JAK/STAT pathway

The **JAK/STAT pathway** is a key signaling mechanism involved in cell growth, differentiation, and immune cell function. In neutrophils, the activation of the JAK/STAT pathway is primarily mediated by cytokines such as **IL-6, IL-17, GM-CSF**, and **IFN-γ**, all of which are upregulated in the TME during tumor progression. Upon cytokine binding to their respective receptors, **JAKs** (Janus kinases) are activated and phosphorylate **STATs** (Signal Transducers and Activators of Transcription), which then translocate to the nucleus and initiate the transcription of target genes involved in neutrophil function.

The molecular effects of the JAK/STAT pathway on neutrophil activity in breast cancer include:

- **Neutrophil activation and survival**: The IL-6/JAK/STAT3 axis is particularly important in promoting neutrophil activation and survival in the TME. STAT3 activation in neutrophils enhances their production of pro-inflammatory cytokines (such as IL-6 and IL-8) and other mediators (e.g., MMPs), which support the tumor’s inflammatory milieu.
- **Chemotaxis**: The IL-17/STAT3 pathway enhances neutrophil recruitment to the TME by inducing the expression of chemokines like CXCL1, CXCL2, and CXCL5 that attract neutrophils to tumor sites. In breast cancer, IL-17 signaling through STAT3 contributes to tumor progression and metastasis by increasing neutrophil infiltration and supporting an inflammatory microenvironment that fosters tumor growth.
- **Pro-tumorigenic polarization**: JAK/STAT signaling is implicated in the polarization of neutrophils toward an **N2-like phenotype**, characterized by the secretion of cytokines and factors that promote tumor growth, angiogenesis, and immune evasion^[73]^.

### MAPK pathway

The **MAPK pathway** encompasses a series of kinases (such as ERK1/2, JNK, and p38 MAPK) that mediate cellular responses to extracellular signals, including stress, inflammation, and cytokine stimulation. In neutrophils, MAPK signaling is triggered by various pro-inflammatory mediators, including TNF-α, IL-1β, CXCL8, and IL-17, leading to the activation of downstream effectors that regulate neutrophil responses such as activation, migration, and degranulation.

The molecular effects of the MAPK pathway on neutrophil activity in breast cancer include:

- **Neutrophil migration and chemotaxis**: MAPK activation, particularly through **ERK1/2** signaling, enhances neutrophil chemotaxis toward tumor-derived chemokines. This is crucial for their recruitment to the TME, where they contribute to the chronic inflammation seen in breast cancer.
- **Neutrophil activation**: MAPK pathways also regulate neutrophil activation by promoting the release of granules containing enzymes and pro-inflammatory mediators (such as **MMPs** and **cytokines**) that facilitate tissue remodeling, metastasis, and immune suppression in the TME.
- **Pro-tumorigenic functions**: In breast cancer, the MAPK pathway may also support the polarization of neutrophils toward a pro-tumorigenic phenotype, where they contribute to tumor growth, invasion, and angiogenesis by secreting factors like VEGF and MMPs^[74]^.

### NF-κB pathway

The NF-κB pathway is a critical mediator of inflammation and immune responses, playing a central role in the activation and function of neutrophils. NF-κB is activated in response to various inflammatory stimuli, such as TNF-α, IL-1β, CXCL8, and PAMPs. Upon activation, the NF-κB dimers (typically p65/p50) translocate to the nucleus and promote the transcription of genes involved in immune responses, inflammation, and cell survival.

The molecular effects of the NF-κB pathway on neutrophil activity in breast cancer include:

- **Neutrophil survival and activation**: NF-κB activation promotes the survival of neutrophils by inhibiting apoptosis and enhancing the secretion of pro-inflammatory cytokines and chemokines, such as CXCL8, TNF-α, and IL-1β, that contribute to a sustained inflammatory environment in the TME.
- **Neutrophil migration**: NF-κB is also involved in the upregulation of adhesion molecules such as Intercellular Adhesion Molecule 1 (**ICAM-1**) and Vascular Cell Adhesion Molecule 1 (**VCAM-1**) on endothelial cells, facilitating neutrophil extravasation and migration into the TME. This recruitment is crucial for establishing a chronic inflammatory microenvironment that aids in tumor progression.
- **Pro-tumorigenic polarization**: In breast cancer, NF-κB activation can lead to neutrophil polarization toward a pro-tumorigenic phenotype (N2-like), which supports tumor growth, metastasis, and angiogenesis through the secretion of factors like VEGF, MMPs, and other matrix-degrading enzymes^[75]^.

## Neutrophil interactions with other immune cells in the tumor microenvironment: implications for breast cancer growth

In the TME, neutrophils interact with various immune cells, including T cells, DCs, and macrophages. These interactions significantly influence the progression and outcome of breast cancer, as neutrophils can either promote or inhibit tumor growth through direct cell–cell contact, the secretion of soluble factors, and the modulation of immune cell activities. The interplay between neutrophils and these other immune cells is complex and multifaceted, with the potential to either foster an anti-tumor immune response or contribute to tumor immune evasion, depending on the context of the TME^[1]^.

### Neutrophil and T cell interactions

T cells are central to the adaptive immune response, and their ability to recognize and destroy tumor cells is a critical factor in controlling cancer progression. Neutrophils interact with T cells in the TME through both direct cell–cell contact and soluble mediators, influencing T cell activation, differentiation, and function.

- **Promotion of tumor growth**: In some instances, neutrophils can promote tumor growth by modulating T cell responses in a way that supports immune tolerance rather than anti-tumor immunity. Neutrophils in the TME can suppress T cell function by releasing immunosuppressive cytokines such as **TGF-β, IL-10**, and **IL-17**, which dampen T cell activation and function. The presence of neutrophils has been shown to promote the differentiation of **regulatory T cells (Tregs)**, which further inhibit effector T cell responses and create a suppressive environment favorable for tumor progression.
- **Inhibition of tumor growth**: Conversely, neutrophils can also exhibit anti-tumor effects by enhancing T cell responses. Neutrophils can present tumor antigens to T cells through **major histocompatibility complex (MHC) molecules** and help prime them for anti-tumor activity. Furthermore, neutrophils can produce **IL-12**, a cytokine that stimulates **CD8 + cytotoxic T cells** to kill tumor cells. Neutrophils can also secrete **IFN-γ**, which enhances the activity of cytotoxic T cells and promotes the expression of **MHC-I molecules** on tumor cells, making them more susceptible to T cell-mediated killing^[2]^.

### Neutrophil and dendritic cell interactions

Dendritic cells are crucial for initiating adaptive immunity by capturing and presenting antigens to T cells. In the TME, DCs can be influenced by neutrophils, which, in turn, affect the subsequent T cell response.

- **Promotion of tumor growth**: Neutrophils in the TME may impair DC function, preventing effective antigen presentation and leading to immune tolerance. Neutrophils can release **TGF-β**, which inhibits DC maturation and their ability to effectively present tumor antigens to T cells. This leads to reduced T cell priming and a failure to mount a robust anti-tumor response. Additionally, neutrophils can influence DCs to adopt a **pro-tumor** phenotype by promoting the secretion of immunosuppressive cytokines like **IL-10** and **IL-4**, further dampening anti-tumor immunity.
- **Inhibition of tumor growth**: On the other hand, neutrophils can enhance DC function by secreting pro-inflammatory cytokines such as **TNF-α** and **IL-1β**, which stimulate DC maturation and their ability to prime naïve T cells against tumor-associated antigens. Neutrophils can also promote the cross-presentation of antigens to CD8 + T cells, further enhancing the anti-tumor immune response^[3]^.

### Neutrophil and macrophage interactions

Macrophages are key players in the immune response within the TME. They can differentiate into **M1 macrophages**, which are pro-inflammatory and have tumor-suppressive functions, or **M2 macrophages**, which are immunosuppressive and promote tumor progression. Neutrophils influence the polarization of macrophages, and this interaction has a significant impact on tumor growth.

- **Promotion of tumor growth**: Neutrophils can promote the polarization of macrophages toward the **M2 phenotype**, which is associated with tumor progression, immune suppression, and angiogenesis. Neutrophils in the TME can secrete **G-CSF** and **IL-17**, which are known to promote M2 macrophage polarization. M2 macrophages then secrete **IL-10** and **TGF-β**, further suppressing immune responses and supporting tumor cell survival and metastasis. Additionally, neutrophils can release **MMPs**, which promote tissue remodeling and enable macrophages to facilitate tumor cell invasion and metastasis.
- **Inhibition of tumor growth**: Alternatively, neutrophils can enhance the anti-tumor immune response by promoting the polarization of macrophages toward the **M1 phenotype**. M1 macrophages are characterized by their ability to produce pro-inflammatory cytokines such as **IL-12, TNF-α**, and **IL-1β**, which enhance the recruitment and activation of T cells and other immune cells to the TME. Neutrophils can secrete **IFN-γ** and **TNF-α**, which activate macrophages and increase their ability to phagocytose tumor cells, present antigens, and secrete cytotoxic factors that inhibit tumor growth^[4,5]^.

### Neutrophil-driven crosstalk and tumor-immune modulation

The interactions between neutrophils and other immune cells in the TME are highly dynamic and subject to modulation by various factors such as cytokines, chemokines, and ECM components. Neutrophils are not only influenced by other immune cells but also play an active role in shaping the immune landscape of the tumor.

- **Neutrophil–tumor cell interaction**: Neutrophils can also directly interact with tumor cells, contributing to the promotion of tumor progression and metastasis. For example, neutrophils can release **MMPs**, which degrade the ECM and promote tumor cell invasion. Additionally, neutrophils can contribute to the formation of the **pre-metastatic niche** by releasing **pro-inflammatory cytokines** that enhance the migration and survival of metastatic tumor cells.
- **Feedback loops**: A crucial aspect of neutrophil-mediated tumor progression is the establishment of feedback loops that sustain the inflammatory microenvironment. Neutrophils secrete a variety of pro-inflammatory mediators that not only recruit additional neutrophils but also modulate the function of other immune cells in the TME. For instance, **IL-17** released by neutrophils can recruit more neutrophils and macrophages, contributing to a chronic inflammatory environment that supports tumor growth. Similarly, neutrophil-derived **TNF-α** and **IL-1β** can further recruit and activate T cells, DCs, and macrophages, which, in turn, amplify the inflammatory response^[6-8]^.

## Polarization of neutrophils in breast cancer: mechanisms leading to pro-tumor (N2) and anti-tumor (N1) phenotypes

Neutrophils, the most abundant leukocytes in the bloodstream, are central to the body’s immune defense against infection and tissue damage. However, in the context of breast cancer, neutrophils exhibit distinct polarization patterns that significantly influence tumor progression. These cells can polarize toward pro-tumor (N2) or anti-tumor (N1) phenotypes, each with different functions in the TME. Understanding the processes that drive this polarization and how these polarized neutrophils influence tumor growth and immune responses is crucial for developing targeted therapies for breast cancer^[4,5]^.

### The N1 phenotype: anti-tumor neutrophils

The N1 phenotype of neutrophils is associated with an anti-tumor response. These neutrophils are pro-inflammatory and are capable of directly attacking tumor cells. They exhibit characteristics that promote the recruitment of other immune cells, enhance the cytotoxicity of T cells, and promote the immune system’s ability to eliminate cancer cells.

#### Mechanisms of N1 polarization

- **Cytokine influence**: The polarization of neutrophils toward the N1 phenotype is primarily driven by pro-inflammatory cytokines such as **IFN-γ, TNF-α**, and **IL-12**. These cytokines are often secreted by activated T cells and NK cells in response to tumor-associated antigens. IFN-γ, in particular, plays a critical role by activating the **JAK/STAT** signaling pathway, which induces the expression of inflammatory genes and enhances neutrophil function.
- **TLR stimulation**: Activation of TLRs, especially **TLR4** and **TLR7**, by PAMPs or damage-associated molecular patterns present in the TME, further promotes N1 polarization. This activation leads to the release of **IL-6, TNF-α**, and other inflammatory cytokines that enhance neutrophil effector functions, including **phagocytosis** and **ROS** production, which contribute to the elimination of tumor cells.
- **NF-κB and MAPK pathways**: The activation of **NF-κB** and **MAPK** signaling pathways in response to inflammatory signals helps sustain the N1 phenotype. These pathways not only regulate the expression of inflammatory cytokines but also influence neutrophil survival, migration, and cytotoxic activity against tumor cells. For instance, **IL-1β** and **TNF-α** signaling through NF-κB can lead to the expression of pro-inflammatory genes that promote tumor cell apoptosis^[6,7]^.

#### Functional characteristics of N1 neutrophils

- **Cytotoxicity**: N1 neutrophils are capable of killing tumor cells through the release of cytotoxic molecules such as **granzyme B** and **perforin**. These molecules directly induce tumor cell death or assist other immune cells, like CD8 + T cells, in recognizing and killing tumor cells.
- **Antigen presentation**: N1 neutrophils can act as antigen-presenting cells (APCs), which further activate the adaptive immune response. They express **MHC** molecules that present tumor antigens to T cells, facilitating the initiation of a robust anti-tumor immune response.
- **Cytokine secretion**: N1 neutrophils secrete pro-inflammatory cytokines such as **IL-12**, which stimulates **CD8 + T cells** and **NK cells**, enhancing their ability to kill tumor cells and promoting an immune response that targets the tumor^[8]^.

### The N2 phenotype: pro-tumor neutrophils

In contrast, the N2 phenotype of neutrophils is associated with tumor promotion. N2 neutrophils possess immune suppressive functions that foster an environment conducive to tumor growth, metastasis, and immune evasion. These cells contribute to chronic inflammation, tissue remodeling, and the recruitment of regulatory immune cells that suppress anti-tumor responses.

#### Mechanisms of N2 polarization

- **Cytokine influence**: The polarization of neutrophils toward the N2 phenotype is driven by the secretion of **TGF-β, IL-4, IL-10**, and **IL-17** in the TME. These cytokines are often produced by tumor cells, tumor-associated macrophages, or regulatory T cells (Tregs) and have a significant role in shaping the immune landscape. **TGF-β** is particularly potent in promoting N2 polarization by inducing the expression of immunosuppressive factors in neutrophils that inhibit the cytotoxic response^[9]^.
- **Angiogenic factors**: The release of angiogenic factors such as **VEGF** by tumor cells can also drive the polarization of neutrophils toward the N2 phenotype. VEGF acts not only to promote blood vessel formation but also to skew the immune response toward a pro-tumor environment by fostering the recruitment of immunosuppressive cells, including neutrophils, and enhancing their survival and activity^[10]^.
- **Chemokine signaling: CXCL1, CXCL2**, and **CXCL8** are key chemokines involved in neutrophil recruitment and polarization in the TME. These chemokines are upregulated in response to tumor cell signaling, promoting neutrophil infiltration into the tumor and the subsequent polarization toward the N2 phenotype. Once in the TME, these neutrophils secrete **MMPs** that degrade the ECM, promoting tumor cell invasion and metastasis^[11]^.
- **PI3K/AKT pathway**: The activation of the **PI3K/AKT** signaling pathway in neutrophils within the TME can enhance their survival and polarization toward an N2 phenotype. This pathway helps neutrophils resist apoptosis, allowing them to persist longer in the TME and continue secreting immunosuppressive cytokines and factors that promote tumor progression^[12]^.

#### Functional characteristics of N2 neutrophils

- **Immune suppression**: N2 neutrophils secrete high levels of **IL-10, TGF-β**, and **IL-17**, cytokines that contribute to immune suppression within the TME. **IL-10** suppresses the activation of T cells and DCs, whereas **TGF-β** promotes the differentiation of regulatory T cells (Tregs), which further dampen the anti-tumor immune response.
- **Tumor promotion**: N2 neutrophils contribute to tumor progression by secreting **MMPs**, which degrade the ECM, thereby enabling tumor cell invasion and metastasis. These neutrophils also produce **VEGF** and **CXCL8**, which promote angiogenesis and recruit additional pro-tumor immune cells, such as macrophages and Tregs, into the TME^[13]^.
- **Tissue remodeling**: The N2 neutrophil phenotype is associated with tissue remodeling and the creation of a pro-inflammatory, pro-TME. N2 neutrophils can help create a niche that supports tumor cell survival, growth, and metastasis by secreting growth factors and remodeling enzymes^[14]^.

### Balance between N1 and N2 phenotypes in breast cancer

The balance between N1 and N2 neutrophil phenotypes in the TME of breast cancer is critical in determining the progression or suppression of the disease. Several factors, including the type of tumor, the presence of specific cytokines, and the composition of the immune landscape, dictate whether neutrophils adopt an N1 or N2 phenotype^[15]^.

- **Pro-inflammatory tumor environment**: In tumors where pro-inflammatory cytokines such as **IFN-γ** and **TNF-α** predominate, neutrophils tend to polarize toward the N1 phenotype, resulting in an anti-tumor immune response. This polarization is often associated with better prognosis and improved survival outcomes in breast cancer patients^[16]^.
- **Immune suppressive tumor environment**: In contrast, tumors that are rich in **TGF-β, IL-10**, and **VEGF** tend to drive neutrophils toward the N2 phenotype, which supports immune evasion and promotes tumor growth. This polarization is often associated with worse prognosis, increased metastatic potential, and resistance to therapy^[17]^.

## The influence of tumor-derived metabolites on neutrophil function in the tumor microenvironment of breast cancer

The TME is a dynamic and complex milieu that plays a crucial role in shaping immune responses, tumor progression, and treatment resistance. In breast cancer, the TME is characterized by various tumor-derived metabolites, which have significant effects on the behavior of immune cells, including neutrophils. Neutrophils, as one of the first responders to inflammation and infection, play a dual role in the TME. They can either promote tumorigenesis or suppress tumor growth, depending on their polarization and the surrounding environmental cues. Tumor-derived metabolites, such as lactate, adenosine, and ketone bodies, influence the function and phenotype of neutrophils, thus impacting tumor progression and immune evasion. Understanding how these metabolites affect neutrophil activity in the TME is crucial for developing novel therapeutic strategies for breast cancer^[18]^.

### Lactate: a key metabolite in tumor progression

Lactate is a well-known byproduct of anaerobic metabolism, and its accumulation in the TME is a hallmark of rapidly proliferating tumors, including breast cancer. The Warburg effect, which describes the preference of cancer cells for aerobic glycolysis even in the presence of oxygen, leads to excessive lactate production. High levels of lactate in the TME have been shown to influence the function of various immune cells, including neutrophils.

#### Impact of lactate on neutrophils

- **Impaired neutrophil function**: Elevated lactate levels in the TME have been shown to impair neutrophil chemotaxis and the ability of neutrophils to respond to infection or inflammation. Lactate interferes with the signaling pathways that govern neutrophil movement, such as the **PI3K/AKT** pathway, thereby reducing their ability to migrate to tumor sites and participate in anti-tumor immune responses^[19]^.
- **Promotion of immunosuppressive phenotype**: Lactate has been implicated in the promotion of an immunosuppressive environment, favoring the polarization of neutrophils toward the **N2** (pro-tumor) phenotype. This phenotype is characterized by the secretion of immunosuppressive cytokines like **IL-10** and **TGF-β**, which dampen the anti-tumor immune response and promote tumor growth. Lactate can activate **HIF-1α** (hypoxia-inducible factor-1 alpha), a transcription factor that regulates the expression of genes involved in immune suppression, further supporting neutrophil polarization toward the N2 phenotype^[20]^.
- **Neutrophil metabolism and ROS production**: Lactate also affects the metabolic profile of neutrophils, shifting their function from oxidative burst and ROS production to a more anti-inflammatory state. This shift in neutrophil function contributes to immune evasion by the tumor, as neutrophils fail to produce the necessary ROS to kill tumor cells effectively^[21]^.

### Adenosine: the role of the adenosine-A2A receptor in neutrophil regulation

Adenosine is another critical metabolite that accumulates in the TME as a result of increased tumor cell proliferation and hypoxia. Adenosine is produced through the breakdown of Adenosine Triphosphate( ATP), and its levels are often elevated in areas of the tumor with high metabolic activity or hypoxic conditions. The effect of adenosine on immune cells, including neutrophils, is primarily mediated through the **A2A adenosine receptor**.

#### Impact of adenosine on neutrophils

- **Suppression of neutrophil function**: Adenosine binding to the **A2A receptor** on neutrophils has been shown to suppress neutrophil activation and function. This includes a decrease in ROS production, reduced degranulation, and impaired phagocytosis, all of which are essential for neutrophil-mediated tumor elimination. Adenosine also inhibits the ability of neutrophils to produce pro-inflammatory cytokines such as **TNF-α** and **IL-1β**, further contributing to an immunosuppressive environment that favors tumor growth.
- **Induction of immunosuppressive phenotype**: Adenosine plays a pivotal role in promoting the polarization of neutrophils toward an N2-like phenotype. This is achieved through the suppression of **NF-κB** activation and the induction of **TGF-β** production, which collectively contribute to an environment that supports tumor progression and immune evasion. The accumulation of adenosine in the TME thus facilitates neutrophil-mediated immune suppression and tumor tolerance^[21,22]^.

### Ketone bodies: metabolic reprogramming and neutrophil function

Ketone bodies, including **β-hydroxybutyrate** (BHB), **acetoacetate**, and **acetone**, are produced during FAO and are important metabolic intermediates that serve as an alternative energy source in nutrient-deprived conditions, such as those often seen in tumors. Recent studies suggest that ketone bodies not only provide metabolic flexibility but also influence immune cell functions, including neutrophils^[23]^.

#### Impact of ketone bodies on neutrophils

- **Enhancement of neutrophil survival**: Ketone bodies, particularly **BHB**, have been shown to enhance neutrophil survival in the TME by modulating their metabolic pathways. This increased survival allows neutrophils to persist longer in the TME, potentially exacerbating their pro-tumor effects in certain contexts. However, in some cases, ketone bodies may help reprogram neutrophils toward a more anti-tumor phenotype by promoting oxidative metabolism and reducing the reliance on glycolysis, which could shift the neutrophil response to a more cytotoxic state^[24]^.
- **Regulation of inflammatory cytokine production**: Ketone bodies have been shown to influence the production of pro-inflammatory cytokines in neutrophils. For example, **BHB** can enhance the production of **IL-12** and **IFN-γ**, cytokines that are critical for promoting Th1 responses and anti-tumor immunity. This suggests that ketone bodies may have a dual role in neutrophil function, either supporting tumor progression or boosting anti-tumor immune responses depending on the metabolic context^[25]^.

### Hypoxia and tumor-derived metabolites

The hypoxic regions of tumors, particularly in larger breast cancers, create a metabolic environment that profoundly affects neutrophil behavior. In these hypoxic conditions, several metabolites, including lactate, adenosine, and certain fatty acids, accumulate and influence neutrophil function^[26]^.

#### Impact of hypoxia on neutrophils

- **HIF-1α activation**: Hypoxia-induced **HIF-1α** (hypoxia-inducible factor 1-alpha) activation in neutrophils plays a key role in shaping their response to the TME. HIF-1α activation under low-oxygen conditions leads to the upregulation of genes involved in immune suppression and tissue remodeling. This further supports neutrophil polarization toward the N2 phenotype, enhancing their role in promoting tumor angiogenesis and metastasis^[27]^.
- **Enhanced immunosuppressive activity**: In hypoxic tumors, neutrophils are often more susceptible to polarization toward an immunosuppressive N2-like state, supported by metabolites such as lactate and adenosine. These metabolites not only hinder the anti-tumor activity of neutrophils but also promote tumor immune escape by suppressing the activation of cytotoxic T cells and NK cells^[28]^.

## Canonical and non-canonical NF-κB activation in neutrophils: roles in the tumor microenvironment of breast cancer

The nuclear factor-kappa B (NF-κB) signaling pathway is a crucial regulator of immune cell function and inflammation. In the context of breast cancer, the NF-κB pathway is often activated within the TME, playing a significant role in the modulation of immune responses and influencing tumor progression. NF-κB activation occurs through two distinct signaling pathways: the canonical and non-canonical pathways. These pathways, although both leading to NF-κB activation, differ in their molecular mechanisms, outcomes, and roles in neutrophil function within the TME. Understanding the distinct contributions of canonical and non-canonical NF-κB activation in neutrophils, and their implications for breast cancer, is critical for developing therapeutic strategies aimed at modulating immune responses in the TME^[76]^.

### Canonical NF-κB pathway in neutrophils within the TME

The canonical NF-κB pathway is primarily activated in neutrophils through the binding of pro-inflammatory cytokines, such as **TNF-α** and **IL-1β**, to their respective receptors. Upon receptor engagement, a series of intracellular signaling events are initiated, ultimately leading to the activation of the NF-κB dimers **p65/RelA** and **p50**. The canonical pathway is predominantly associated with inflammation and immune responses, and its activation plays a pivotal role in the neutrophil response to breast cancer.

#### Functions of canonical NF-κB activation in neutrophils

- **Inflammatory cytokine production**: Activation of the canonical NF-κB pathway in neutrophils promotes the expression of key inflammatory cytokines and chemokines such as **CXCL1, CXCL2, IL-8**, and **TNF-α**, which recruit and activate other immune cells in the TME. These molecules enhance neutrophil chemotaxis, support the recruitment of other immune cells (such as T cells and macrophages), and sustain a pro-inflammatory environment in the tumor^[50]^.
- **Neutrophil survival and activation**: Canonical NF-κB activation also enhances neutrophil survival by upregulating anti-apoptotic proteins like **Bcl-2** and **cFLIP**. This ensures the persistence of neutrophils within the TME, enabling them to continue their immune surveillance activities and contribute to the chronic inflammation seen in many cancers, including breast cancer^[72]^.
- **ROS production and tumor killing**: The canonical pathway is involved in the regulation of neutrophil effector functions, such as ROS production and degranulation. These activities are essential for the ability of neutrophils to kill tumor cells directly. ROS production, in particular, helps neutrophils mediate oxidative damage to both cancer cells and the ECM, thereby supporting tissue remodeling and potentially limiting tumor spread^[73]^.
- **Contribution to tumor progression**: While canonical NF-κB activation enhances anti-tumor responses, it can also contribute to tumor progression when dysregulated. Prolonged NF-κB activation in neutrophils within the TME can lead to sustained inflammatory responses, fostering tumor angiogenesis, invasion, and metastasis. By producing pro-inflammatory cytokines like **TNF-α** and **IL-1β**, neutrophils can enhance tumor cell proliferation and survival, thereby indirectly supporting tumor growth^[73]^.

### Non-canonical NF-κB pathway in neutrophils within the TME

The non-canonical NF-κB pathway is a more specialized signaling pathway, primarily activated by members of the **TNF receptor superfamily**, such as **BAFF** (B-cell activating factor), **RANKL** (receptor activator of NF-κB ligand), and **CD40**. This pathway predominantly activates the **p52**/RelB dimers, as opposed to the canonical **p65/RelA** complex, and leads to the modulation of a different set of genes that are typically involved in immune cell differentiation, survival, and tissue remodeling. Non-canonical NF-κB signaling has a distinct impact on neutrophil function in the TME, contributing to immune regulation and influencing tumor progression in subtle ways^[74]^.

#### Functions of non-canonical NF-κB activation in neutrophils

- **Regulation of neutrophil differentiation and polarization**: Non-canonical NF-κB activation in neutrophils has been implicated in the regulation of their differentiation and polarization. Specifically, it has been suggested that non-canonical NF-κB signaling can influence the balance between the pro-inflammatory **N1 phenotype** and the immunosuppressive **N2 phenotype** of neutrophils. Non-canonical signaling pathways could promote the polarization of neutrophils toward the N2-like phenotype, which is often associated with chronic inflammation, tissue remodeling, and enhanced tumor progression in breast cancer^[74]^.
- **Tissue remodeling and angiogenesis**: Non-canonical NF-κB activation in neutrophils plays a significant role in tissue remodeling and the regulation of angiogenesis within the TME. Neutrophils activated through non-canonical NF-κB signaling are capable of secreting a variety of MMPs and growth factors, including **VEGF**. These factors contribute to the formation of new blood vessels, which are critical for tumor growth and metastasis. Thus, non-canonical NF-κB signaling in neutrophils may indirectly promote tumor progression by supporting angiogenesis and enhancing the metastatic potential of cancer cells^[74]^.
- **Immune suppression**: Similar to its role in polarization, non-canonical NF-κB activation in neutrophils can also promote immune suppression in the TME. Neutrophils with activated non-canonical NF-κB pathways are more likely to produce immunosuppressive cytokines, such as **TGF-β** and **IL-10**, which dampen anti-tumor immunity and facilitate immune evasion by the tumor. This shift toward an immunosuppressive environment allows the tumor to evade immune surveillance, leading to an increased risk of metastasis and treatment resistance^[75]^.
- **Regulation of neutrophil-mediated inflammation**: Non-canonical NF-κB signaling also affects the regulation of chronic inflammation in neutrophils. By modulating the secretion of inflammatory mediators, non-canonical NF-κB activation in neutrophils can contribute to the long-lasting inflammatory state often seen in cancer. Chronic inflammation within the TME not only promotes tumor progression but also creates a favorable environment for the survival and growth of cancer stem cells, which are known to be resistant to many conventional therapies.

### Functional differences between canonical and non-canonical NF-κB pathways in neutrophils

The functional distinctions between the canonical and non-canonical NF-κB pathways in neutrophils are critical for understanding their roles in breast cancer progression:

- **Pro-inflammatory vs. immunosuppressive functions**: Canonical NF-κB activation is generally associated with pro-inflammatory functions, supporting tumor immune surveillance and potentially limiting tumor growth. In contrast, non-canonical NF-κB activation tends to promote immune suppression, polarization of neutrophils toward the N2 phenotype, and tumor-promoting processes like angiogenesis and tissue remodeling.
- **Acute vs. chronic inflammation**: Canonical NF-κB activation is typically involved in acute inflammatory responses, such as the initiation of immune responses to infection or injury. However, its chronic activation in the TME may contribute to sustained inflammation and tumor progression. On the other hand, non-canonical NF-κB activation is more involved in chronic inflammation, immune regulation, and tissue remodeling, processes that are crucial for the establishment and progression of tumors in the TME.
- **Tumor-supportive roles**: Both canonical and non-canonical NF-κB signaling contribute to tumor-supportive roles but through different mechanisms. Canonical signaling supports neutrophil-mediated tumor killing and immune responses, while non-canonical signaling promotes angiogenesis, immune suppression, and the development of an immune-evasive TME^[74,76]^.

## Substances released by tumor-associated neutrophils (TANs) that prevent T-cell activation and effector function in breast cancer

Tumor-associated neutrophils (TANs) are a prominent component of the TME and can play a crucial role in modulating immune responses in breast cancer. While neutrophils are typically thought of as mediators of the innate immune response against infections and tumors, within the TME, TANs often contribute to immune evasion rather than tumor suppression. A major mechanism through which TANs dampen anti-tumor immunity is the release of various substances, including **TGF-β, IL-10**, and **ROS**. These molecules can directly or indirectly impair the activation and effector function of T-cells, thus hindering the body’s ability to mount an effective anti-tumor immune response in breast cancer^[37]^.

### TGF-β and its role in T-cell inhibition

TGF-β is a potent immunosuppressive cytokine that is frequently secreted by TANs within the TME. This cytokine plays a critical role in shaping the immune landscape of the tumor and is often associated with immune suppression and cancer progression.

#### Mechanism of TGF-β-mediated T-cell suppression

- **Inhibition of T-cell activation**: TGF-β suppresses the activation of naïve T-cells by inhibiting the expression of key co-stimulatory molecules such as **CD28** on T-cells and **B7** on APCs. This results in a reduced T-cell response to tumor antigens, weakening the ability of T-cells to detect and eliminate cancer cells. Additionally, TGF-β impairs the expression of T-cell receptors (TCRs), further preventing effective antigen recognition and activation.
- **Induction of Treg cells**: TGF-β can also drive the differentiation of **regulatory T-cells (Tregs)**, which are known for their immunosuppressive functions. Tregs, in turn, suppress the activation and function of effector T-cells (CD8 + cytotoxic T-cells and CD4 + helper T-cells), thereby diminishing the immune system’s ability to mount an anti-tumor response. In breast cancer, TGF-β-induced Tregs contribute to a pro-tumor environment by inhibiting anti-tumor immunity.
- **Impaired cytokine production**: TGF-β further impairs T-cell function by inhibiting the production of crucial cytokines, such as **IFN-γ**, that are necessary for effective T-cell effector function. This inhibition leads to a reduced cytotoxic response against tumor cells, allowing cancer cells to escape immune surveillance and continue to proliferate^[37]^.

### IL-10 and its role in T-cell suppression

IL-10 is another cytokine released by TANs that contributes to immune evasion in breast cancer. Although IL-10 has anti-inflammatory properties that can prevent excessive tissue damage, its release in the TME is often associated with immune suppression and tumor progression.

#### Mechanism of IL-10-mediated T-cell inhibition

- **Downregulation of T-cell activation**: IL-10 suppresses T-cell activation by inhibiting the expression of co-stimulatory molecules on APCs and T-cells. IL-10 reduces the expression of **CD80** and **CD86** on APCs, which are critical for providing the second signal required for T-cell activation. As a result, even if T-cells encounter APCs in the TME, their ability to become activated and mount an immune response is significantly diminished^[77]^.
- **Induction of Tregs**: Similar to TGF-β, IL-10 promotes the expansion of Tregs in the TME. Tregs, which secrete additional immunosuppressive factors like **IL-35** and **TGF-β**, contribute to a feedback loop of immune suppression. By increasing Treg numbers, IL-10 further reduces the effectiveness of cytotoxic T-cells (CD8 + T-cells) and helper T-cells (CD4 + T-cells), weakening the immune response against breast cancer cells.
- **Impairment of Th1 response**: IL-10 has been shown to inhibit the **Th1-type immune response**, which is essential for the activation of CD8 + T-cells and the production of pro-inflammatory cytokines like **IFN-γ**. In breast cancer, this suppression of Th1 responses limits the ability of effector T-cells to respond to the tumor, allowing it to grow unchecked^[37]^.

### ROS and its role in T-cell dysfunction

Reactive oxygen species (ROS) are chemically reactive molecules produced by TANs as part of their immune response, especially in the context of inflammation. While ROS are generally thought to help neutrophils kill pathogens and tumor cells, excessive or dysregulated ROS production within the TME can lead to significant immune dysfunction.

#### Mechanism of ROS-mediated T-cell inhibition

- **Oxidative damage to T cells**: ROS are capable of inducing oxidative stress in nearby T-cells, leading to cellular damage, DNA mutations, and apoptosis. This oxidative stress not only impairs the survival of T-cells but also affects their ability to proliferate and function properly in the TME. Specifically, ROS-induced damage can reduce the activation of TCRs and limit the ability of T-cells to produce cytokines such as **IFN-γ**, which are essential for their anti-tumor function^[77]^.
- **Impairment of T-cell metabolism**: The production of ROS in the TME also interferes with the metabolic pathways required for T-cell activation and function. T-cells rely on metabolic shifts (such as the Warburg effect) to support their activation and effector functions. ROS can disrupt these metabolic pathways, particularly by inhibiting the mitochondrial oxidative phosphorylation necessary for energy production. This impairment diminishes the ability of T-cells to mount a robust anti-tumor response.
- **Induction of exhaustion in T cells**: In addition to causing oxidative damage, ROS can contribute to the development of **T-cell exhaustion**, a state of dysfunction that is commonly seen in chronic infections and cancer. Exhausted T-cells exhibit reduced proliferative capacity, diminished cytokine production, and impaired cytotoxicity. In the TME, ROS production by TANs can drive T-cells into this exhausted state, effectively rendering them unable to recognize and kill breast cancer cells^[77]^.

### Cooperation of TGF-β, IL-10, and ROS in modulating T-cell function

The substances released by TANs – TGF-β, IL-10, and ROS – work together in a coordinated manner to suppress T-cell activation and function in the TME. This collaboration creates an immunosuppressive environment that hinders the body’s ability to mount an effective anti-tumor immune response in breast cancer.

- **TGF-β and IL-10 synergy**: Both TGF-β and IL-10 induce the expansion of Tregs, which further exacerbate the suppression of effector T-cells. Additionally, IL-10’s inhibition of the Th1 response and TGF-β’s inhibition of T-cell activation complement each other, creating a dual mechanism for blocking the anti-tumor immune response.
- **ROS enhancement of immunosuppression**: ROS-induced oxidative stress enhances the immunosuppressive activity of TGF-β and IL-10 by promoting T-cell exhaustion and DNA damage. In this way, ROS not only directly impairs T-cell function but also indirectly facilitates a more immune-evasive TME through the promotion of Treg expansion and the inhibition of T-cell metabolism [82,83.

## Enhancing anti-tumor effects by combining neutrophil-targeted and checkpoint inhibitors with targeted therapies, chemotherapy, or radiation in breast cancer

The battle between the immune system and cancer cells is often complex and multifaceted. In breast cancer, the TME consists of a variety of immune cells, stromal cells, and signaling molecules, all of which work together to either promote or inhibit tumor progression. Neutrophils, particularly TANs, play a crucial role in shaping the TME and influencing tumor progression. While neutrophils can exert anti-tumor effects, they can also support tumor growth and metastasis by contributing to immune suppression and chronic inflammation. This dual role of neutrophils suggests that targeting them therapeutically could be a promising strategy for breast cancer treatment. Combining neutrophil-targeted therapies with ICIs, as well as conventional treatment options such as chemotherapy, radiation, and targeted therapies, holds significant potential for improving anti-tumor effects and clinical outcomes^[37]^.

### Neutrophil-targeted therapies in breast cancer

The targeting of neutrophils in the TME for therapeutic purposes is an emerging area of research in breast cancer. Neutrophils contribute to the tumor’s immune evasion by releasing pro-inflammatory cytokines and chemokines, as well as creating an immunosuppressive microenvironment. Strategies aimed at modulating neutrophil function or depleting TANs could help to re-establish anti-tumor immune responses and prevent tumor progression.

#### Targeting neutrophil recruitment and activation

One approach to manipulating neutrophil activity in the TME is to block the recruitment and activation of neutrophils through the inhibition of key chemokines and receptors. For instance, the chemokine receptor **CXCR2** plays a pivotal role in neutrophil recruitment to sites of inflammation and tumors. Targeting **CXCR2** with specific antagonists or antibodies could reduce the number of TANs in the TME and, consequently, diminish the pro-tumor effects of these cells. By limiting neutrophil infiltration, this approach could decrease the secretion of pro-inflammatory cytokines and reduce tumor-promoting chronic inflammation. Furthermore, **CD11b** is a surface receptor on neutrophils that is involved in their adhesion to the endothelium during migration. Targeting **CD11b** could not only inhibit neutrophil recruitment but also block their activation within the TME, potentially improving the anti-tumor immune response^[77]^.

#### Modulating neutrophil phenotypes

Neutrophils can exist in distinct phenotypic states within the TME, particularly pro-tumor (N2) and anti-tumor (N1) phenotypes. While N1 neutrophils exhibit anti-tumor properties through the secretion of pro-inflammatory cytokines and ROS, N2 neutrophils promote tumor progression by supporting angiogenesis and metastasis, as well as suppressing T-cell activation. Modulating neutrophil polarization toward the anti-tumor N1 phenotype is an appealing strategy for enhancing anti-tumor immunity. Therapeutic agents that influence signaling pathways, such as the **PI3K/AKT, MAPK**, or **NF-κB** pathways, could be used to push neutrophils toward the N1 phenotype. Additionally, using cytokines like **IFN-γ** or **GM-CSF**, which have been shown to induce an anti-tumor neutrophil phenotype, could help skew neutrophils toward a pro-inflammatory state that promotes tumor cell killing^[78]^.

### Checkpoint inhibitors in breast cancer therapy

ICIs have revolutionized the treatment landscape for a variety of cancers, including breast cancer, by blocking the inhibitory signals that dampen T-cell activity. Key immune checkpoint proteins such as **PD-1, PD-L1**, and **CTLA-4** are critical in maintaining immune tolerance and preventing autoimmunity. However, cancer cells exploit these pathways to evade immune surveillance, thus allowing the tumor to grow unchecked. In breast cancer, the use of **anti-PD-1** and **anti-PD-L1** antibodies has shown promise in some subtypes of the disease, particularly in TNBC. These checkpoint inhibitors work by blocking the interaction between PD-1 on T-cells and PD-L1 on tumor cells or APCs, thereby reactivating T-cell responses and allowing them to attack the tumor. However, while immune checkpoint blockade has shown clinical benefit in breast cancer, many patients either do not respond or eventually develop resistance. This resistance could be due, in part, to the immunosuppressive TME, where TANs contribute to immune evasion by releasing cytokines such as **TGF-β** and **IL-10**, which inhibit T-cell activation and promote the expansion of regulatory T-cells (Tregs). Therefore, combining neutrophil-targeted therapies with checkpoint inhibitors may enhance the effectiveness of immunotherapy in breast cancer^[79,80]^.

### Combining neutrophil-targeted therapies with checkpoint inhibitors, chemotherapy, and radiation

To improve the efficacy of current breast cancer treatments, there is a growing interest in combining neutrophil-targeted therapies with other therapeutic modalities such as ICIs, chemotherapy, and radiation. The rationale behind this approach lies in the complementary mechanisms of action of these therapies, which can work synergistically to overcome the challenges posed by the TME.

#### Neutrophil-targeted therapies and checkpoint inhibitors

Combining neutrophil-targeted therapies with ICIs holds great potential for enhancing anti-tumor immunity. For example, targeting neutrophils to reduce their suppressive activity in the TME could help restore T-cell function, making immune checkpoint blockade more effective. By reducing the immunosuppressive effects of TANs, this combination therapy could improve T-cell activation and increase the likelihood of a durable anti-tumor response. Additionally, neutrophil-targeted therapies could help prevent or overcome resistance to checkpoint inhibitors. As TANs are involved in creating an immune-evasive environment, reducing their influence could restore immune responsiveness to checkpoint inhibition, potentially enhancing the effectiveness of ICIs in breast cancer patients who have not previously responded^[81]^.

#### Neutrophil-targeted therapies and chemotherapy

Chemotherapy remains a cornerstone in the treatment of breast cancer, particularly for aggressive subtypes such as TNBC. While chemotherapy has been shown to reduce tumor burden, it can also promote inflammation and tumor-promoting signaling pathways that enhance neutrophil recruitment and activation. Combining neutrophil-targeted therapies with chemotherapy could limit these pro-tumor effects by reducing the recruitment of neutrophils to the tumor site and preventing their pro-inflammatory signaling. Furthermore, neutrophils can influence the development of chemotherapy resistance by promoting tumor cell survival through the secretion of cytokines and growth factors. Targeting neutrophils in combination with chemotherapy could reduce this resistance and enhance the efficacy of treatment^[82]^.

#### Neutrophil-targeted therapies and radiation

Radiation therapy is another essential treatment modality for breast cancer, particularly in patients with localized tumors or after surgical resection. However, radiation can also induce inflammation and promote the recruitment of neutrophils to the irradiated tumor area. While neutrophils can contribute to tumor control through their cytotoxic properties, excessive neutrophil activation and ROS production can also lead to tissue damage and tumor progression. Combining neutrophil-targeted therapies with radiation could help mitigate the harmful effects of radiation-induced inflammation. By reducing neutrophil infiltration or reprogramming neutrophils to an anti-tumor phenotype, this combination therapy could enhance the therapeutic effects of radiation while reducing tumor recurrence and metastasis^[81,82]^.

## Biomarkers for targeted treatment in breast cancer: the role of CXCR2, CD66b, and neutrophil-to-lymphocyte ratio (NLR)

Breast cancer, a heterogeneous disease, has a complex TME that plays a pivotal role in tumor progression, metastasis, and treatment resistance. Immune cells, particularly neutrophils, are key players in this environment. While neutrophils can exert anti-tumor effects, they can also promote tumor growth and immune suppression, depending on their phenotype and activation status. As a result, identifying reliable biomarkers to predict neutrophil behavior and tailor treatment strategies could improve therapeutic outcomes in breast cancer. Certain biomarkers, including **CXCR2, CD66b**, and the **NLR**, are currently being explored for their potential to guide treatment and assess prognosis in breast cancer patients^[83]^.

### CXCR2 as a prognostic biomarker in breast cancer

**CXCR2** is a chemokine receptor that plays a crucial role in neutrophil recruitment and activation. In the context of breast cancer, CXCR2 is implicated in neutrophil infiltration within the TME, where it is involved in promoting inflammation, angiogenesis, and metastasis. The binding of CXCR2 to its ligands, such as **CXCL1, CXCL2,** and **CXCL8**, results in neutrophil chemotaxis and activation, which can foster a pro-tumor environment. Elevated expression of CXCR2 has been observed in various cancer types, including breast cancer, where it is linked to poor prognosis and increased metastatic potential. Recent studies have shown that high levels of CXCR2 expression on neutrophils in the TME correlate with an increased number of TANs and a more aggressive breast cancer phenotype. Furthermore, CXCR2 + neutrophils in the TME contribute to immune evasion by promoting the accumulation of immunosuppressive cells, such as regulatory T-cells (Tregs), and inhibiting effective anti-tumor immune responses. Given its role in neutrophil recruitment and tumor progression, CXCR2 is being actively studied as a potential biomarker for predicting treatment response and guiding therapeutic strategies. Targeting CXCR2 or its ligands could serve as an effective therapeutic strategy to limit neutrophil infiltration and reduce the immunosuppressive environment, potentially enhancing the effectiveness of other treatments, such as ICIs. Thus, measuring CXCR2 expression levels in breast cancer patients may help identify those most likely to benefit from therapies targeting the neutrophil-mediated immune response^[84]^.

### CD66b as a marker of neutrophil activation in breast cancer

**CD66b**, a glycoprotein expressed on the surface of neutrophils, is a marker of neutrophil activation and degranulation. CD66b plays a role in promoting neutrophil adhesion, migration, and the formation of NETs, which are implicated in tumor progression and metastasis. In breast cancer, CD66b has been associated with the activation of neutrophils in the TME, where it can contribute to an inflammatory microenvironment conducive to tumor growth. Elevated levels of CD66b expression on circulating neutrophils have been linked to poor prognosis in various cancers, including breast cancer. The presence of activated neutrophils in the peripheral blood, as indicated by CD66b expression, correlates with increased tumor burden, metastasis, and a higher likelihood of recurrence. Thus, CD66b can serve as a valuable biomarker for assessing neutrophil activation and predicting the aggressiveness of breast cancer. Furthermore, CD66b can potentially be used to monitor the effectiveness of therapies that target neutrophil activation or neutrophil-mediated inflammation, providing clinicians with a tool for personalized treatment^[85]^.

### Neutrophil-to-lymphocyte ratio (NLR) as a prognostic indicator

The NLR is an easily measurable biomarker that reflects the balance between neutrophils and lymphocytes in the peripheral blood. It is a non-invasive marker of systemic inflammation and immune status, and its elevated levels are often associated with poor prognosis in various cancers, including breast cancer. An increased NLR indicates a predominance of neutrophils relative to lymphocytes, which is typically reflective of a pro-inflammatory state that can foster tumor progression. In breast cancer, high NLR has been linked to advanced disease stages, increased metastatic potential, and reduced survival. Neutrophils, in particular, are known to promote cancer progression through the release of pro-inflammatory cytokines, ROS, and enzymes that degrade the ECM, facilitating tumor invasion and metastasis. Elevated NLR suggests an increased neutrophil presence in the circulation and, by extension, a more pro-tumor environment within the TME. On the other hand, a low NLR indicates a more favorable immune environment with a stronger lymphocytic response, which is associated with better clinical outcomes. Given its simplicity and cost-effectiveness, NLR is being explored as a tool to predict prognosis and guide treatment decisions in breast cancer. It can be easily obtained from routine blood tests and may help clinicians identify patients who are at higher risk of poor outcomes or those who may benefit from more aggressive or immunologically focused treatments. Additionally, NLR could be used as a monitoring tool to assess the effectiveness of therapies targeting neutrophils or inflammation, such as those combined with ICIs or chemotherapy^[83]^.

## Implications for targeted therapies and clinical practice

The exploration of biomarkers such as CXCR2, CD66b, and NLR holds significant promise for improving breast cancer treatment. By identifying patients with high neutrophil activity or a pro-inflammatory TME, clinicians could better tailor treatment strategies to target the underlying inflammatory processes that drive tumor progression. For example, patients with high CXCR2 expression or elevated NLR might benefit from therapies that target neutrophil recruitment or reduce systemic inflammation. These could include small molecule inhibitors of CXCR2, which have shown promise in preclinical studies and early-phase clinical trials. Additionally, combining neutrophil-targeted therapies with ICIs, chemotherapy, or radiation could help overcome the immunosuppressive effects of TANs and enhance the overall anti-tumor immune response. Furthermore, CD66b expression could be used as a marker to assess neutrophil activation in real-time, enabling the monitoring of treatment efficacy and adjustments to therapeutic strategies. In combination with other immune markers, CD66b could provide a comprehensive view of the immune status of the patient, aiding in the personalization of cancer therapies^[85-87]^.

## Conclusion

The role of neutrophils in breast cancer progression is complex and multifaceted. Neutrophils can have both pro-tumor and anti-tumor effects depending on their activation status and the signaling pathways involved. Understanding how these immune cells interact with other components of the TME and contribute to tumor growth, metastasis, and immune evasion is crucial for improving therapeutic strategies. Biomarkers like **CXCR2, CD66b,** and **NLR** have emerged as valuable tools to better understand the dynamics of neutrophil behavior in breast cancer and to guide treatment decisions. These markers not only provide insights into the inflammatory landscape of the TME but also have the potential to predict treatment responses and patient outcomes, making them valuable for clinical practice.

However, there are still many challenges in translating these findings into clinical practice. While targeting neutrophil-mediated inflammation has shown promise in preclinical studies, further research is needed to fully understand the nuances of neutrophil polarization, the impact of different neutrophil subsets, and their interactions with other immune cells. Moreover, combining neutrophil-targeted therapies with other modalities, such as ICIs, chemotherapy, and radiation, requires a deeper understanding of the underlying mechanisms driving immune suppression within the TME. Future research should focus on exploring how neutrophils contribute to breast cancer heterogeneity, particularly in metastatic disease. Longitudinal studies that assess the temporal dynamics of neutrophil infiltration and activation during treatment would be crucial for identifying early predictive biomarkers of treatment response and resistance. Additionally, investigating the role of neutrophil-targeted therapies in combination with novel immune-modulating agents and evaluating their effects in clinical trials will be essential to determine the efficacy and safety of such treatments.

## Data Availability

Not applicable as this is a review.

## Research registration unique identifying number (UIN)

Not applicable as this is a review.
